# Accuracy, Reliability and Waveform-Level Agreement Between the Impera Force Platform and a Criterion-Standard Force Plate

**DOI:** 10.3390/s26185850

**Published:** 2026-09-15

**Authors:** Dario Pompa, Alessandra Caporale, Antonio Buglione, Pietro Picerno, Johnny Padulo

**Affiliations:** 1 Department of Medicine and Aging Sciences, University “G. d’Annunzio” of Chieti-Pescara, 66100 Chieti, Italy; dario.pompa@unich.it; 2 BIND—Behavioral Imaging and Neural Dynamics Center, University “G. d’Annunzio” of Chieti-Pescara, 66100 Chieti, Italy; 3 Department of Neuroscience, Imaging and Clinical Sciences, University “G. d’Annunzio” of Chieti-Pescara, 66100 Chieti, Italy; alessandra.caporale@unich.it; 4 Institute of Advanced Biomedical Technologies (ITAB), 66100 Chieti, Italy; 5 Department of Theoretical and Applied Sciences, eCampus University, 22060 Novedrate, Italy; 6 School of Pharmacy, University of Camerino, 62032 Camerino, Italy; pietro.picerno@unicam.it; 7 Department of Biomedical Sciences for Health, Università degli Studi di Milano, 20134 Milan, Italy; johnny.padulo@unimi.it

**Keywords:** force platform, countermovement jump, squat jump, waveform-level agreement, test–retest reliability, ground reaction force

## Abstract

Laboratory-grade force platforms are the reference standard for quantifying ground reaction forces but remain costly and confined to well-resourced laboratories, limiting field-based use. This study evaluated the accuracy and between-session reliability of a portable force platform (Impera, Spinitalia) under static loading, and its concurrent validity during vertical jumping, against a criterion-standard piezoelectric plate with calibration traceable to national standards (4Jump, Kistler). Accuracy and between-session reliability were assessed against five tared loads (21.6–103.2 kg) applied at five plate locations across two sessions 24 h apart. Concurrent validity was assessed during squat and countermovement jumps performed by ten physically active men, using intraclass correlation coefficients, Bland–Altman analysis and the linear fit method applied to the entire force–time waveform. Both platforms underestimated the lightest load by about 4% and approached zero error at higher loads; the between-device difference was 0.19 kg and did not translate into a difference in proportional accuracy (*p* = 0.151), with no device × position interaction. Between-session coefficients of variation were below 1% for both devices. Concurrent agreement was good to excellent for all jump variables (ICC 0.867–0.996), and waveform agreement was close to unity (R^2^ ≥ 0.997), with about 2% amplitude underestimation, greater in participants generating the highest impact forces. The Impera platform provides accurate and reliable measurements under static loading, and force–time data that agree closely with the 4Jump during vertical jump testing.

## 1. Introduction

In strength and conditioning practice, the objective assessment of neuromuscular function underpins athlete monitoring and the individualisation of training [1]. The vertical ground reaction force recorded by a force platform is widely regarded as the reference method for this purpose during jumping, since outcomes such as jump height (JH), impulse, power and the reactive strength index are derived directly from the force–time signal [2,3]. Beyond these outcomes, force plates resolve the drivers and strategy of the movement, such as braking and propulsive forces and phase durations, allowing practitioners to establish whether an athlete produces sufficient force in the time available and to target the qualities that require development [4]. The bilateral countermovement jump (CMJ) is the most frequently used test for monitoring neuromuscular function, owing to its time efficiency and low resource demands, and is commonly complemented by the squat jump (SJ) to probe concentric force production [3,5].

Criterion-standard force plates, typically built around piezoelectric or strain-gauge transducers, provide the accuracy and sampling rates required for these assessments, but their cost and the expertise required to operate and maintain them restrict access largely to well-resourced laboratories [2]. This constraint has driven the recent proliferation of low-cost, portable alternatives, ranging from smartphone-based video analysis and contact mats to compact load-cell platforms, extending jump monitoring to field settings where a criterion-standard system is not a realistic option [4,6,7,8].

The practical value of any such device depends entirely on evidence that it measures what it is meant to measure. Before a portable platform can replace, or complement, a criterion-standard reference, its accuracy against known physical quantities, its reliability across repeated sessions, and its concurrent validity against a criterion device all need to be established. Reliability is commonly quantified with the coefficient of variation and with the intraclass correlation coefficient, whose form and interpretation should be explicitly reported given the several variants of this coefficient that are available [9,10,11]. Concurrent agreement is typically examined with the Bland–Altman approach, which characterises the bias and the limits of agreement between two methods of measurement rather than relying on correlation alone [12]. These variable-level statistics, however, summarise each derived outcome, such as JH or peak force, in isolation and can conceal disagreement in the shape of the underlying signal; comparing the entire force–time waveform, for instance with the linear fit method originally developed for gait kinematic and kinetic curves [13], gives a more complete picture of whether a device reproduces the mechanical event under study rather than only a handful of derived numbers [14]. Recent reviews have nonetheless highlighted substantial heterogeneity and a lack of uniformity in metric definitions, calculations and phase terminology across force-plate studies, which currently limits comparison between investigations and underscores the need for standardised, well-characterised measurement [5].

Prior validations of portable and low-cost force measurement systems have largely addressed these criteria in isolation and at the level of discrete variables. For jumping, portable plates have been compared with criterion-standard reference systems for CMJ force–time variables, showing near-perfect correlations, trivial biases and limits of agreement of around 2% [1,4]; however, these comparisons were confined to the CMJ, were limited to discrete variables without a waveform-level analysis, and did not include a static accuracy check against known loads [1,4], while reliability was assessed either within-session only [4] or across different floor surfaces rather than against known masses [1]. More recent work has extended this approach to wireless dual-plate systems: the hardware of one such system was compared with an in-ground criterion plate during the CMJ and drop jump using ordinary least products regression, with no fixed or proportional bias for any CMJ variable [8], and another dual-plate system was validated against a piezoelectric criterion plate for CMJ variables [15] and for CMJ performance more generally [2]. A commercially available portable plate has likewise been examined for both reliability and validity during the CMJ [7]. In all of these studies, agreement was established between discrete outcome and strategy variables, and none included an assessment of accuracy against known masses. A contact mat validated against a force platform for CMJ and SJ height alone proved not interchangeable [16], and a portable plate compared with a criterion-standard device across several tasks was tested in only two participants and without between-session reliability or waveform analysis [17]. For isometric testing, a low-cost plate and load cell have been examined for peak force and rate of force development, yet explicitly as an inter-device agreement analysis rather than against a criterion-standard [18]. Reviews of applied force-plate use similarly note that most evidence originates from fixed laboratory settings and call for greater attention to reliability and field application [3].

The most complete appraisal of a low-cost platform to date combined static linearity and regional uniformity against known weights placed across nine regions of the sensing surface with a simultaneous dynamic comparison against a criterion-standard plate during squat, countermovement and drop jumps, and additionally characterised the frequency response of the platform by impact testing [6]. That study reported excellent static linearity and trivial regional differences, and establishes the template that the present work follows most closely. Its dynamic comparison, however, as in all the validations cited above, rested on correlations between discrete variables.

Force platforms of this type are already being used to collect force–time data in applied elite settings and in peer-reviewed research [19], including the platform examined in the current study, which makes an independent metrological characterisation of their performance all the more necessary. What has rarely been examined for any portable platform is whether the shape of the force–time curve itself is faithfully reproduced, and whether it is preserved across the functionally distinct phases of a jump, from submaximal propulsion to impact loading.

Validating field-based tools against these accuracy, reliability and validity criteria is a well-established research line, and the same rigour has previously been applied by our group to a range of sport-science instruments, including a standalone GNSS receiver for outdoor running [20], an isometric bench for knee assessment [21], and a smartphone application for vertical JH [22]. The present study extends this framework to a new portable force platform.

The aim of this study was to determine, in a single design combining static and dynamic loading conditions, the accuracy of the Impera platform, a portable four-load-cell system developed for research use and not currently marketed as commercial product, with reference to five tared masses, its between-session test–retest reliability under static loading, together with its concurrent validity, at both the variable and the full waveform level, against a criterion-standard reference force plate (4Jump, Kistler) during the SJ and the CMJ. The present study combines these established validation criteria with an analysis of the entire force–time waveform and, separately, of the functionally distinct phases of each jump, which allows the agreement between devices to be examined where it is most likely to differ. We hypothesised that, under static loading, the tested platform would show measurement errors of a magnitude similar to those of the reference device and acceptable between-session reliability, and that it would show good-to-excellent concurrent validity for the main kinetic variables and for the force–time waveform of both jump tasks.

## 2. Materials and Methods

### 2.1. Study Design

**Figure 1:**
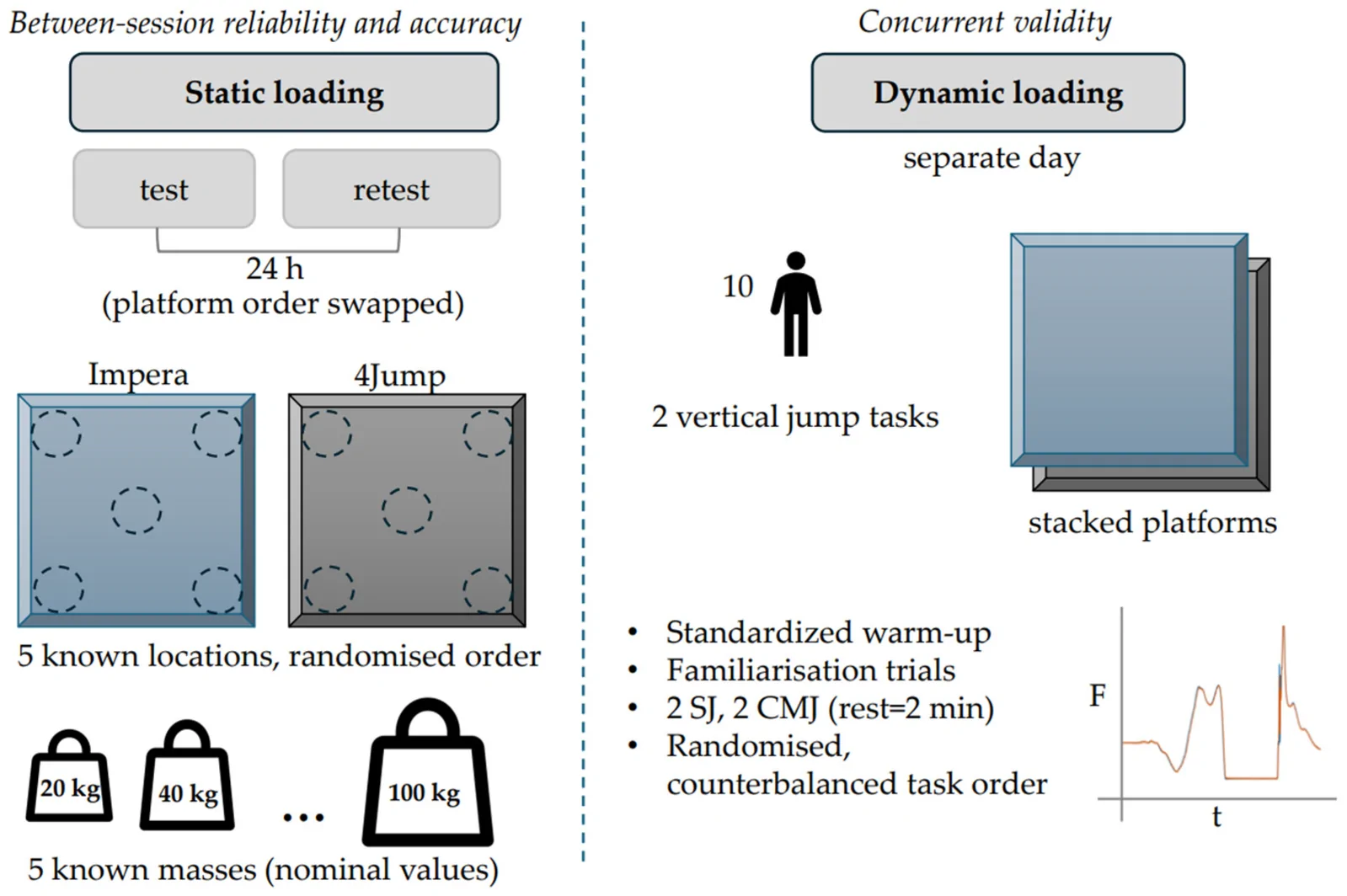
Schematic of the experimental procedure. Data collection comprised three sessions. The first two were static loading sessions, separated by 24 h and identical in content, to evaluate test–retest reliability and accuracy. In each of these sessions five known masses were applied at five locations on each of the two platforms, tested sequentially with one measurement per load × location combination, and with the platform order reversed in the second session. The third session, carried out on a separate day, was a dynamic loading session, performed to assess concurrent agreement between the two platforms. In this session the Impera platform was positioned on top of the 4Jump, so that both devices recorded simultaneously, while ten participants performed two squat jumps (SJ) and two countermovement jumps (CMJ) each, in randomised counterbalanced order with 2 min of rest between consecutive trials.

A cross-sectional validation study was conducted to evaluate the accuracy, test–retest reliability and concurrent agreement of the Impera force platform against a criterion-standard reference force plate under static and dynamic loading conditions. The study comprised two protocols, a static and a dynamic one: in the former, the accuracy of each platform was assessed against five known masses, and its between-session test–retest reliability was assessed across two recording sessions; in the latter, the concurrent agreement of the Impera platform with the reference plate was assessed during two ballistic vertical-jump tasks recorded simultaneously by both devices. Test–retest reliability was therefore assessed for static loading only; the dynamic protocol was designed to establish concurrent validity and did not involve repeated sessions. Data collection comprised three sessions in total: two static sessions, separated by 24 h and identical in content, and a dynamic session carried out on a separate day (Figure 1).

The study was conducted in accordance with the Declaration of Helsinki and approved by the Ethics Committee of the University of Milan (protocol code 104/25; date of approval 14 October 2025). All participants provided written informed consent prior to participation.

### 2.2. Participants

Ten physically active participants (all males; age: 24.2 ± 1.7 years) took part in the dynamic protocol. Prior to testing, each participant’s body mass and stature were recorded (body mass: 72.3 ± 5.2 kg; stature: 1.81 ± 0.06 m). All participants were free from musculoskeletal injury at the time of testing and were familiar with the jump tasks. No formal a priori sample size estimation was performed. The number of participants was determined by the paired within-subject design, in which each jump is recorded simultaneously by both devices, so that between-device agreement is assessed without the biological variability that would affect separate trials. The static protocol did not involve human participants, being based on the application of known masses to the two platforms.

### 2.3. Instrumentation

Two force platforms were compared. The force platform Impera (Spinitalia, Pomezia, Italy) is a portable system that samples, with its four load sensors (one at each corner), the vertical ground reaction force at up to 1000 Hz and streams data in real time to a personal computer through the manufacturer’s software (Spinitalia Lab Version 9.6.4.3). The sensing elements are spoke-type (wheel-type) strain-gauge load cells (model YGX-DL102; Shenzhen Yi Guan Xing Tech Development Co., Ltd., Shenzhen, China), each with a rated capacity of 500 kg (4905 N per cell; because the usable capacity of the assembled platform depends on how the load is distributed across the four cells, the per-cell rating rather than their sum defines the operating limit). Each cell is configured as a full Wheatstone bridge, with an input impedance of 750 ± 20 Ω, an output impedance of 700 ± 5 Ω and a recommended excitation of 5–15 V. According to the manufacturer’s specifications, the rated output is 2.0 ± 0.05 mV/V, with non-linearity, hysteresis and repeatability each ≤ ±0.03% of full scale (FS), creep over 30 min ≤ ±0.03% FS, zero balance ≤ ±1% FS, and temperature effects on both zero and output ≤ ±0.03% FS per 10 °C over an operating range of −20 to +80 °C. The cells withstand a safe overload of 150% FS and are rated IP67.

Signals from the four cells were conditioned by purpose-developed amplifiers and digitised by a dedicated five-channel acquisition board, with four channels assigned to the load cells and a fifth channel available for auxiliary synchronised inputs, such as a linear position transducer. The board is powered directly through the USB connection to the host computer, requires no external power supply, and provides analogue output channels reproducing the load cell signals both before and after amplification. The board acquires at 10 k-samples/s per channel, and the vertical ground reaction force was recorded at 1000 Hz by averaging each group of ten consecutive samples. The assembled platform measures 770 × 770 × 120 mm^2^ and weighs 12 kg, and the signal is digitised at 12 bits. These values are nominal manufacturer specifications, and those reported above for non-linearity, hysteresis, repeatability and creep refer to the individual load cells as supplied by their manufacturer rather than to the assembled platform, whose integrated performance is not characterised in the available documentation; the natural frequency of the assembled platform is not declared by the manufacturer. The present study provides an independent assessment of the assembled system under both static and dynamic loading.

The reference device 4Jump was a criterion-standard piezoelectric force plate (Kistler Type 9290DD; Kistler Instrumente AG, Winterthur, Switzerland), which measures the vertical component of the ground reaction force (one force dimension) over a measurement range of 0–10 kN, with an overload limit of 15 kN, a linearity of <±0.5% of full-scale output (FSO), a hysteresis of <1% FSO and a natural frequency of approximately 150 Hz. The plate measures 920 × 920 × 125 mm, weighs 21.6 kg and acquires the vertical ground reaction force at 500 Hz with a resolution of 14 bits. The specific reference unit used in the present study was calibrated by the manufacturer using a continuous calibration, comparison method against a working standard (Kistler 96017633; Kistler Instrumente AG, Winterthur, Switzerland) and a precision calibrator (Kistler 5395B; Kistler Instrumente AG, Winterthur, Switzerland), at an ambient temperature of 23 °C and a relative humidity of 45%. Over the 0–10 kN range the calibration returned a sensitivity of 1.003 N/N and a linearity including hysteresis of ±0.19% FSO, with a calibration and measurement capability (CMC; coverage factor k = 2, corresponding to a coverage probability of approximately 95%) of 0.20%; over the 0–1 kN range the corresponding values were 1.002 N/N, ±0.24% FSO and 0.25%. The calibration certificate fulfils the requirements of EN 10204 inspection certificate 3.1, and the reference equipment is traceable to national standards. All forces recorded in the present study fell within the calibrated 0–10 kN range.

Both systems transmitted data to a dedicated personal computer running the corresponding acquisition software (MARS Quattro Jump Installer 5.2.1.0237 for the reference plate). For the dynamic comparison, the Impera platform signal was down sampled from 1000 Hz to 500 Hz within the manufacturer’s software (Spinitalia Lab) to match the sampling frequency of the reference plate. Downsampling was verified to consist of simple decimation, with every second sample retained and no low-pass filtering applied; spectral analysis of the impact phase showed that less than 0.01% of the signal energy lay above 250 Hz, so aliasing was negligible.

### 2.4. Experimental Procedures

#### 2.4.1. Static Protocol

**Figure 2:**
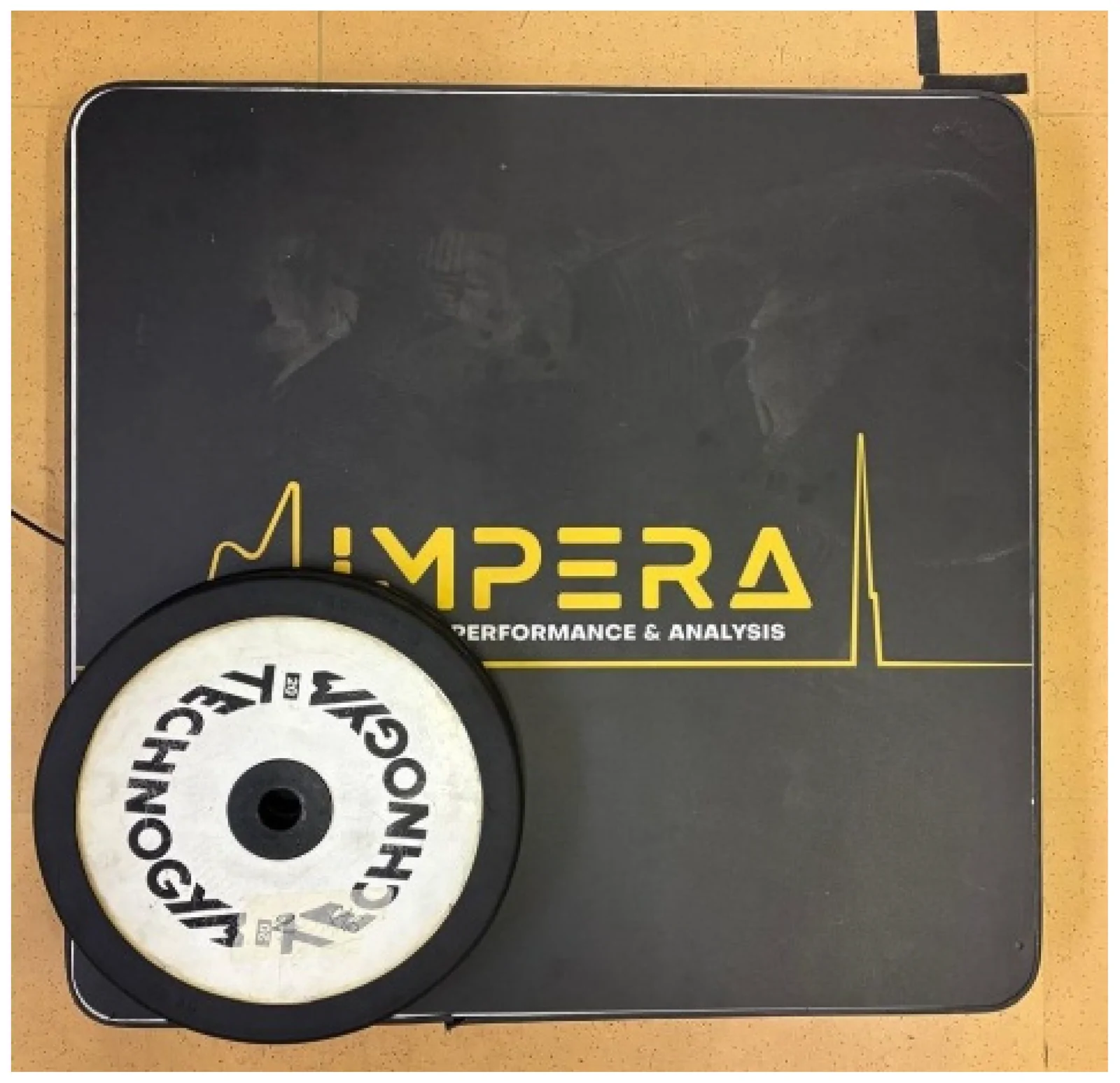
Known mass positioned in a corner of the Impera platform.

Known masses were generated using tared discs (four discs labelled 20 kg and two discs labelled 10 kg; Technogym S.p.A., Cesena, Italy; Figure 2). Each disc was weighed individually on a mechanical column scale (ASIMED MB301, Aparatos y Sistemas de Medida S.A., Barcelona, Spain; serial no. 6321; Max 150 kg, Min 2 kg, verification scale interval e = 100 g, accuracy class III) to determine its true mass and was uniquely labelled to ensure that the same discs were used in every test. The discs were combined into five cumulative loads spanning approximately 20 to 100 kg, corresponding to measured masses of 21.6, 41.9, 62.5, 82.3 and 103.2 kg. Both systems were switched on and allowed to stabilise for approximately 30 min, after which each platform was zeroed under no-load conditions before each session. The two platforms were tested sequentially rather than simultaneously, each load being applied to one platform at a time, and the order in which the two platforms were tested was reversed in the second session. Each load was applied separately to each platform at five locations on the plate surface (centre and the four corners), in randomised order, and the procedure was repeated across two sessions separated by 24 h. Each load × location combination was measured once per session on each platform, yielding 25 measurements per platform in each session and 100 measurements in total. For each measurement, the load was held static for approximately 10 s and the recorded value was extracted as the mean force over the stable portion of that holding interval, excluding the loading and unloading transients, and the load was removed and repositioned between consecutive measurements.

#### 2.4.2. Dynamic Protocol

**Figure 3:**
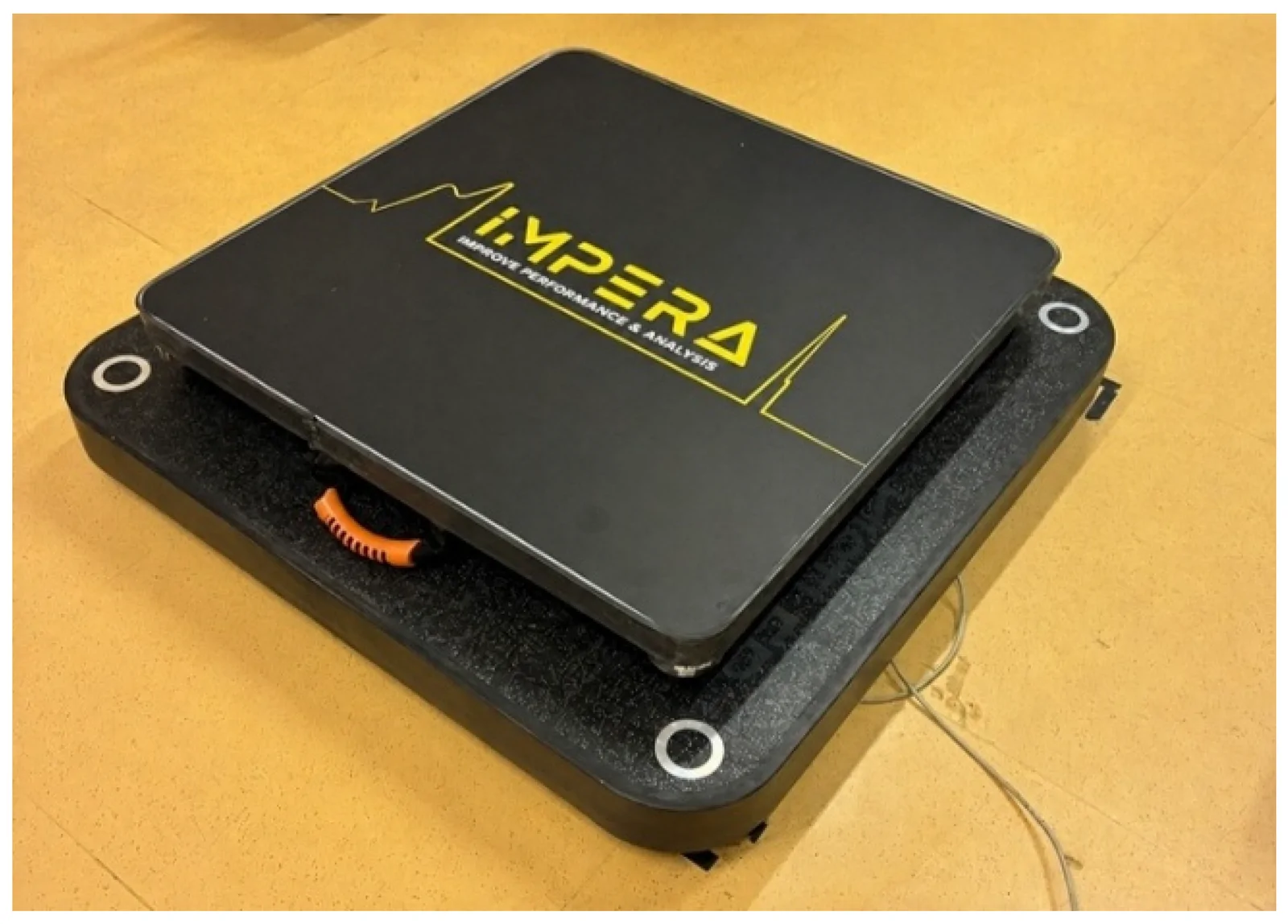
The Impera platform positioned on top of the 4Jump force plate.

Participants performed two vertical-jump tasks: the SJ and CMJ. To enable simultaneous acquisition, the 4Jump was positioned on the floor and the Impera platform was placed directly on top of it, so that both devices captured each jump concurrently (Figure 3), an arrangement previously used to compare force platforms recording the same trial [2,4,6]. The reference plate is the larger of the two (920 × 920 mm^2^ versus 770 × 770 mm^2^), so this was the only possible arrangement. Both systems were switched on and allowed to stabilise for approximately 30 min. With the two platforms in their final stacked configuration and with no participant standing on them, both devices were then zeroed before each session; for the reference plate, this procedure also excluded the mass of the overlying Impera platform from the measured body weight.

Following a standardised warm-up [23] and two familiarisation trials, each participant performed two SJ and two CMJ with hands on the hips. Consecutive trials were separated by a rest interval of 2 min, and the order of the two jump tasks was randomised and counterbalanced across participants. The SJ was initiated from a static semi-squat position (knee angle of approximately 90°) held for about 3 s, with no preliminary countermovement, so that propulsion was produced through a purely concentric action. The CMJ was initiated from an upright standing position and consisted of a rapid downward (eccentric) movement immediately followed by an explosive upward (concentric) push. For each participant, the trial with the higher JH, as measured by the reference plate, was retained for analysis, so that the comparison was performed on the trial with the greatest propulsive impulse and take-off velocity. Because both devices recorded the same jump simultaneously, this selection could not bias the between-device comparison. From the vertical force–time signal recorded by each device, the following variables were derived: JH [m], mean braking and mean propulsive force [N], braking and propulsive impulse [N·s], mean propulsive power expressed relative to body mass [W·kg^−1^], braking and propulsive time [s], and RSI_MOD_ [a.u.] [24,25]. Movement onset, take-off, and the braking and propulsive phases were identified using the same velocity-based approach and custom software previously described [24] applied here to the centre-of-mass velocity derived from the vertical force–time signal of each device. For the SJ, only propulsive-phase variables were computed, as this task does not include a braking (countermovement) phase. Body mass used for normalisation was derived separately for each device, from the mean vertical force recorded during the quiet standing period preceding each trial, so that the two processing chains remained fully independent. Post-processing was performed with a custom application written in C++ (Microsoft Visual Studio 2022, Microsoft, Redmond, WA, USA), which computed the force–time signals and extracted the jump variables uniformly across all trials [24].

### 2.5. Statistical Analysis

All analyses were performed on raw values in JASP (version 0.97.0; JASP Team, Amsterdam, The Netherlands). Statistical significance was set at α = 0.05. For each platform, accuracy under static loading was expressed as the absolute error (measured value minus theoretical value) and as the percentage error relative to the known mass:

(1) $$e_{i}\;=\;F_{\text{meas},i}\;-\;F_{\text{ref},i}$$

(2) $$e_{\%,i}=\frac{e_{i}}{F_{\text{ref},i}}\times 100$$

where F_meas,i_ is the value measured by the platform in the i-th observation and F_ref,i_ the corresponding theoretical value.

Descriptive statistics (mean with 95% confidence interval, standard deviation, minimum and maximum) were computed for both error metrics. The effects of load, device and plate location on static accuracy were examined using two separate three-way mixed-effects analyses of variance (ANOVAs), one for the absolute error and one for the percentage error, with load (five levels) and device (Impera versus 4Jump) and plate location (centre and four corners) as fixed factors and Type III sums of squares. Each condition was measured in two sessions, yielding 100 observations in total. Because the two sessions were repeated measurements of the same devices rather than independent replicates, session was modelled as a random intercept, with the models estimated by restricted maximum likelihood and fixed-effect tests based on Satterthwaite degrees of freedom. With only two sessions, the between-session variance component is estimated with a single degree of freedom and therefore with low precision; between-session variability is quantified directly in the reliability analysis rather than through this model. Each load × device × location cell was measured once per session, so the residual stratum is composed of the within-cell between-session differences and all fixed effects are tested against the test–retest variability of the instruments. Any between-session component therefore inflates the residual mean square and reduces the F-statistics, so this specification cannot inflate the Type I error rate. Because the design was fully balanced, Type II and Type III sums of squares were equivalent. Effect sizes were reported as partial eta-squared ($\eta_{p}^{2}$), computed as the ratio of the effect sum of squares to the sum of the effect and residual sums of squares:

(3) $$\eta_{p}^{2}\;=\;\frac{{SS}_{\text{effect}}}{{SS}_{\text{effect}}\;+\;{SS}_{\text{error}}}$$

where SS_effect_ is the sum of squares of the effect of interest and SS_error_ the residual sum of squares.

Eta-squared values, expressing each effect as a proportion of the total variance, are reported in the Supplementary Materials (Table S1). Effect sizes were interpreted alongside the corresponding differences expressed in physical units, since a large standardised effect may still correspond to a difference too small to matter in practice. Normality of residuals was assessed with the Shapiro–Wilk test. With only two observations per cell, formal tests of variance homogeneity were uninformative, and homogeneity was therefore evaluated by inspecting the spread of residuals across cells. Residuals were normally distributed for the absolute error (W = 0.977, *p* = 0.075) but departed from normality for the percentage error (W = 0.868, *p* < 0.001), where residual variability scaled inversely with load magnitude, as expected when an approximately constant absolute error is expressed relative to a varying denominator. Given the fully balanced design, the F-test is robust to this departure, and the absolute error was therefore treated as the primary metric, with percentage-error results interpreted alongside those for the absolute error. Significant main effects were followed by Tukey-corrected post hoc comparisons.

Plate location is of specific interest for a platform based on four independent load cells and was therefore included as a third factor in both ANOVAs. Position-specific error values for each device are reported in Appendix B (Table A5 and Table A6).

Between-session (session 1 versus session 2) test–retest reliability of the static measurements was quantified separately for each platform using the coefficient of variation (CV%), computed as:

(4) $$CV\%\;=\;\frac{SD}{\bar{x}}\;\times \;100$$

where SD and $\bar{x}$ are, respectively, the standard deviation and the mean of the two session values, reported as the mean with 95% confidence interval. Reliability was complemented by Bland–Altman analysis comparing the two sessions, with the between-session difference plotted against the mean of the two sessions, reporting the bias (mean difference between sessions) and the 95% limits of agreement (bias ± 1.96 SD), each with its 95% confidence interval. The bias and the limits of agreement were computed as:

(5) $$\bar{d}\;=\;\frac{1}{n}\sum_{i=1}^{n}d_{i}$$

(6) $$\mathrm{LoA}=\bar{d}\;\pm \;1.96\;s_{d}$$

where d_i_ is the difference between the two paired measurements of the i-th observation, n the number of paired observations, $\bar{d}$ the bias and s_d_ the standard deviation of the differences.

Concurrent agreement between Impera and the 4Jump plate was assessed separately for the SJ and the CMJ. For each jump variable, the intraclass correlation coefficient (two-way random-effects model, absolute agreement, single measures; ICC_2,1_ as defined by Shrout and Fleiss [9]) was computed with its 95% confidence interval, with the two devices treated as the two measurements. The ICC was computed as:

(7) $$ICC\left(2,1\right)\;=\;\frac{{MS}_{R}\;-\;{MS}_{E}}{{MS}_{R}\;+\;\left(k\;-\;1\right){MS}_{E}\;+\;\left(\frac{k}{n}\right)\left({MS}_{C}\;-\;{MS}_{E}\right)}$$

where MS_R_ is the mean square for rows (participants), MS_C_ the mean square for columns (devices), MS_E_ the residual mean square, k the number of devices (k = 2) and n the number of participants. ICC values were interpreted as *poor* (<0.50), *moderate* (0.50–0.75), *good* (0.75–0.90) and *excellent* (>0.90) [10]. Agreement was further examined using Bland–Altman analysis, reporting the bias (mean difference, 4Jump minus Impera) and the 95% limits of agreement (bias ± 1.96 SD), computed as in Equations (5) and (6), each with its 95% confidence interval, for every variable (individual plots are provided in the Supplementary Materials). To allow comparison across variables expressed in different units, the bias and the half-width of the limits of agreement were additionally expressed as a percentage of the mean of the two devices. 

Beyond the variable-level agreement described above, the concurrent agreement of the entire vertical force–time waveform of the SJ and the CMJ was quantified using the linear fit method [13]. Both signals were analysed at 500 Hz (see Section 2.3). For each participant, the two force–time signals were first coarsely aligned on the peak force of the landing phase, which is the sharpest transient of the trial and therefore the most reliable common feature. Residual misalignment was then removed by maximising the normalised cross-correlation between the two signals within a window centred on that peak, searching over a range of ±10 samples (±20 ms at 500 Hz); the lag returned by this procedure was applied by shifting the Impera signal accordingly, so that all subsequent fitting was performed on fully aligned samples. The residual lag and the maximum cross-correlation coefficient are reported alongside the fit parameters as indicators of synchronisation quality. Because the regression was fitted to signals that had already been aligned, the resulting R^2^ quantifies waveform similarity conditional on alignment and does not itself constitute evidence of inter-device synchronisation; the latter is quantified solely by the residual lag. A first-order linear regression was then fitted between the two aligned signals, taking the reference force as the independent variable and the Impera force as the dependent variable, and yielding the amplitude coefficient a_1_ (the slope), the offset a_0_ (the intercept), the coefficient of determination R^2^ (interpreted as a waveform-similarity index) and the root-mean-square deviation (RMSD); the signal range of each device was also computed. The regression model and the associated indices were defined as:

(8) $$F_{Imp}\left(t\right)\;=\;a_{1}F_{ref}\left(t\right)\;+\;a_{0}$$

(9) $$R^{2}=1-\frac{{SS}_{res}}{{SS}_{tot}}$$

(10) $$RMSD=\sqrt{\frac{\sum_{t=1}^{N}{\left(F_{Imp}\left(t\right)-{\hat{F}}_{Imp}\left(t\right)\right)}^{2}}{N\;-\;2}}$$

where F_ref_(t) and F_Imp_(t) are the aligned force samples of the reference and the Impera platform at sample t, $\hat{F}$_Imp_(t) is the value predicted by Equation (8), N the number of samples in the analysis window, a_1_ the amplitude coefficient, a_0_ the offset, SS_res_ the residual sum of squares and SS_tot_ the total sum of squares about the mean of F_Imp_.

Consistent with the linear fit method, both R^2^ and a_1_ are expected to approach unity, values close to 1 denoting identical waveform shape and amplitude; a_0_ quantifies the residual offset; and the linearity assumption is regarded as valid when R^2^ > 0.50. The relationship between the amplitude coefficient and the magnitude of the forces sampled was examined by correlating a_1_ with the reference signal range across participants, separately for each jump task and for the whole trial and each analysis window. These computations were performed in MATLAB (version 2025a; The MathWorks, Natick, MA, USA), and the resulting parameters were summarised across participants as the mean ± standard deviation.

In addition to the whole-trial analysis, the same linear fit procedure was applied separately to two functionally distinct phases of each trial. Phase boundaries were identified on the reference signal, and the corresponding sample indices were applied to both devices, which were aligned sample-by-sample after synchronisation. This isolates waveform agreement within corresponding phases from any difference in phase detection between devices, and the phase-specific analysis therefore does not itself validate the ability of the Impera platform to identify phase boundaries. Body weight was estimated from the last stable window preceding take-off. The propulsion window extended from movement onset to take-off in the SJ, and from the onset of braking, defined as the instant of minimum centre-of-mass velocity, to take-off in the CMJ. The landing braking window extended from touchdown to the instant at which vertical force returned to body weight, so that both windows were delimited by the same physical criterion. Take-off was defined as the first sample falling below a 20 N [26] threshold after the propulsive peak, and touchdown as the first sample returning above that threshold after the flight phase. Phase-specific results are reported in Appendix A (Table A1, Table A2, Table A3 and Table A4).

## 3. Results

### 3.1. Static Accuracy

Both platforms measured the five tared loads with small errors. The percentage error followed the same load-dependent pattern in the two devices (Table 1): both plates underestimated the lightest load (≈−4% at 21.6 kg), the error decreased in magnitude as the load increased, and it approached zero, turning slightly positive, at the two heaviest loads. Averaged across loads, the mean error was −0.078 kg (95% CI −0.263, 0.108) for the 4Jump and −0.269 kg (95% CI −0.427, −0.111) for the Impera in absolute terms, and −0.861% (95% CI −1.373, −0.349) and −1.019% (95% CI −1.491, −0.548), respectively, in percentage terms. In both models the between-session variance component was estimated at the boundary of the parameter space (σ^2^ = 0), indicating that variability between sessions was not distinguishable from residual measurement variability. Both models returned a singular fit. A between-session difference in mean error of 0.046 kg would have been required to produce a positive variance estimate in the absolute-error model, against the 0.002 kg observed; the corresponding threshold for the percentage-error model was 0.108%, against 0.074% observed. The three-way mixed-effects ANOVAs indicated that measurement error was determined primarily by the applied load, which exerted a large effect on both the absolute and the percentage error (Table 2). Plate location also affected error, but with a substantially smaller effect size, whereas the effect of device was negligible: small but significant for the absolute error and non-significant for the percentage error. Critically, the device × location interaction was not significant for either error metric, providing no evidence that the two platforms responded differently to load placement; position-specific values for each device are reported in Appendix B (Table A5 and Table A6). A load × device interaction was present for the absolute error but not for the percentage error, indicating that the modest difference between devices varied slightly across loads while no corresponding difference emerged in relative terms. A load × position interaction was marginally significant for the absolute error only (*p* = 0.043), reflecting slightly different position-related deviations across load levels.

### 3.2. Between-Session Test–Retest Reliability (Static Protocol)

**Figure 4:**
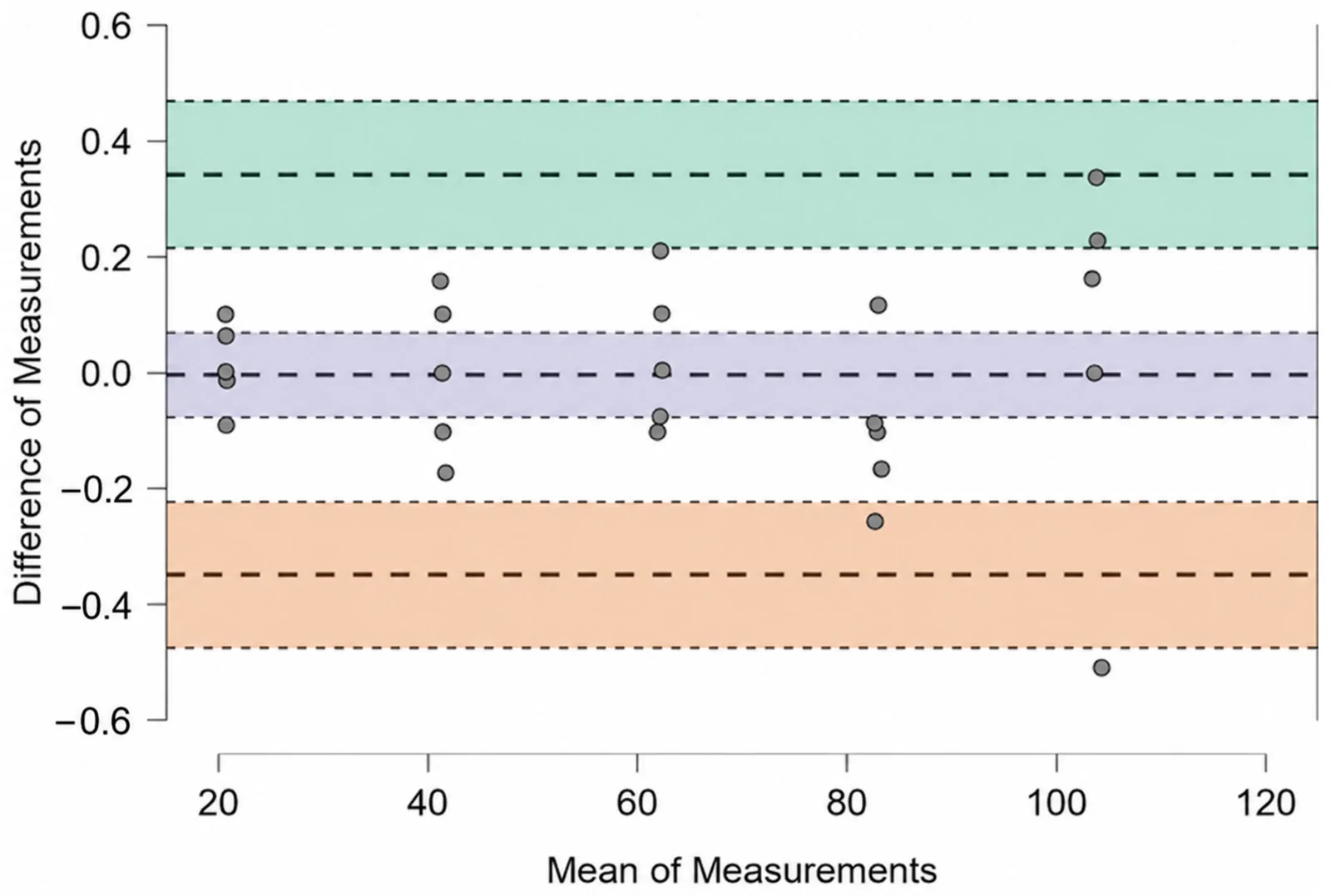
Bland–Altman Plot for the 4Jumps (t1 vs. t2). The two weighting sessions (t1, t2) were performed 24 h apart. Both axes are expressed in kilograms (kg): the mean of measurements (x-axis) and the difference of measurements (y-axis). The central dashed line represents the mean difference (bias), and the upper and lower dashed lines represent the 95% limits of agreement. The purple-coloured band represents the 95% confidence interval around the bias, whereas the green- and orange-coloured bands represent the 95% confidence interval for the upper and lower limit of agreement, respectively. The short-dashed lines delimit these confidence intervals.

**Figure 5:**
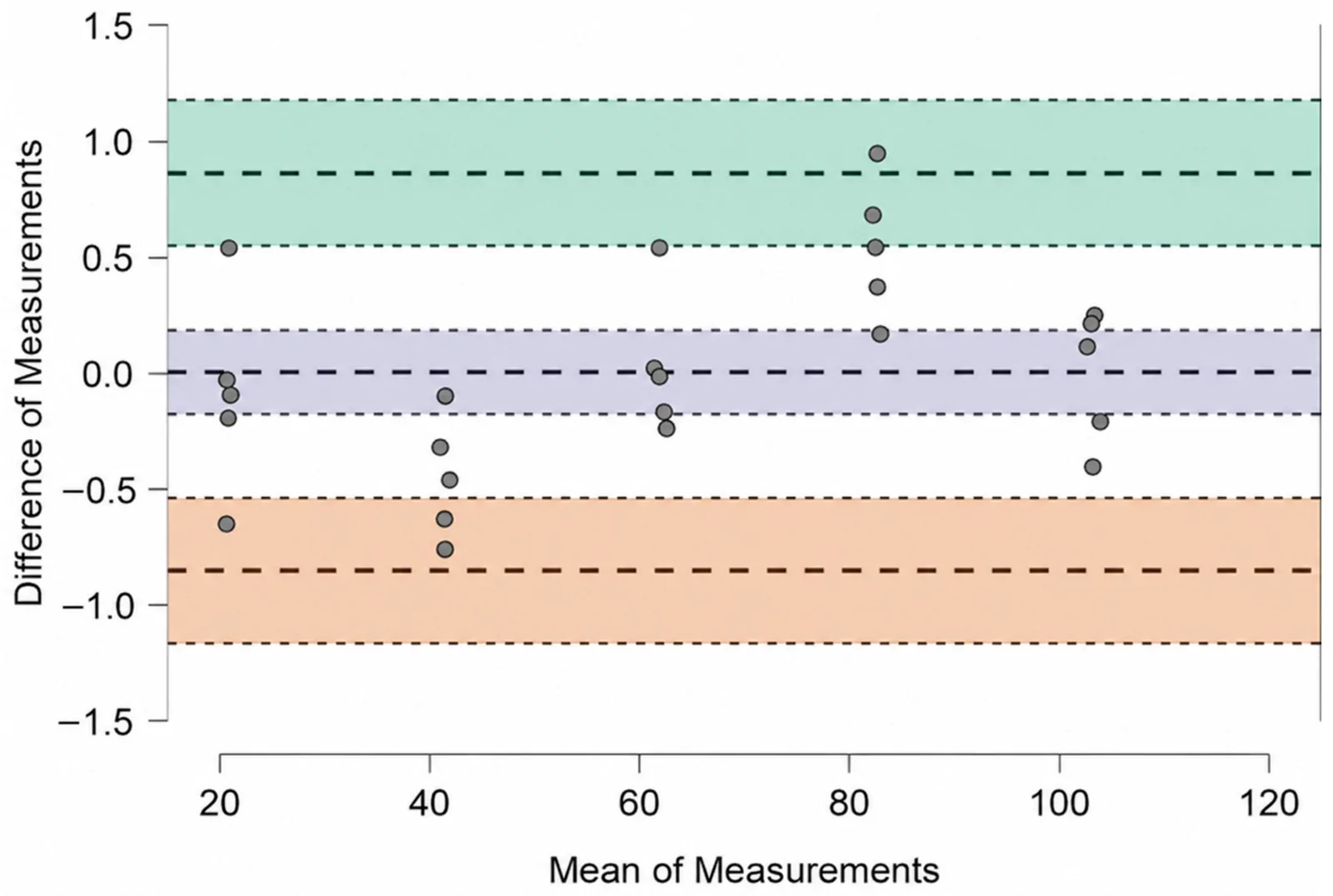
Bland–Altman Plot for the Impera (t1 vs. t2). The two weighting sessions (t1, t2) were performed 24 h apart. Both axes are expressed in kilograms (kg): the mean of measurements (x-axis) and the difference of measurements (y-axis). The central dashed line represents the mean difference (bias), and the upper and lower dashed lines represent the 95% limits of agreement. The purple-coloured band represents the 95% confidence interval around the bias, whereas the green- and orange-coloured bands represent the 95% confidence interval for the upper and lower limit of agreement, respectively. The short-dashed lines delimit these confidence intervals.

The measurements from both platforms were highly repeatable between the two sessions, with coefficients of variation below 1% and a near-zero between-session bias bounded by narrow limits of agreement (Table 3; Figure 4 and Figure 5). Reliability was higher for the reference plate, whose coefficient of variation and limits of agreement were narrower than those of the Impera by approximately three-fold and 2.5-fold, respectively (0.155% versus 0.530%; ±0.35 kg versus ±0.86 kg), although both devices remained below 1% variability.

### 3.3. Concurrent Agreement Between Devices

Concurrent agreement between the Impera and the reference plate was good to excellent for both jump tasks. For the CMJ, agreement was excellent (ICC_2,1_ > 0.90) for seven of the nine variables, JH and propulsive impulse showing good agreement; for the SJ, agreement was excellent for all variables except JH and propulsive impulse, both of which showed good agreement. For the braking and propulsive force and time variables, for braking impulse and propulsive power in the SJ, point estimates were ≥ 0.976 with lower confidence limits above 0.90, so excellent agreement can be asserted with confidence. For RSI_MOD_ in the SJ and propulsive power in the CMJ, the lower limits were 0.826 and 0.850 respectively, indicating agreement that is at least good. For jump height and propulsive impulse in both tasks and for RSI_MOD_ in the CMJ, the intervals were considerably wider and their lower limits fell between 0.577 and 0.739, so the data are compatible with agreement ranging from moderate to excellent and the point-estimate classification should not be read as established. Point estimates and 95% confidence intervals for each variable are reported in Table 4.

Agreement was further examined using Bland–Altman analysis (reference minus Impera). The bias did not exceed 3.2% of the mean for any variable and was positive for all propulsive-force variables. The limits of agreement were wider than the bias for every variable, and were narrowest for the force and time variables and widest for jump height and RSI_MOD_. Bias, limits of agreement and the corresponding means are reported in Table 5. The complete Bland–Altman plots for the CMJ and the SJ are provided in the Supplementary Materials (Figures S1–S9 and S10–S15, respectively).

### 3.4. Force–Time Waveform Agreement (Linear Fit Method)

**Figure 6:**
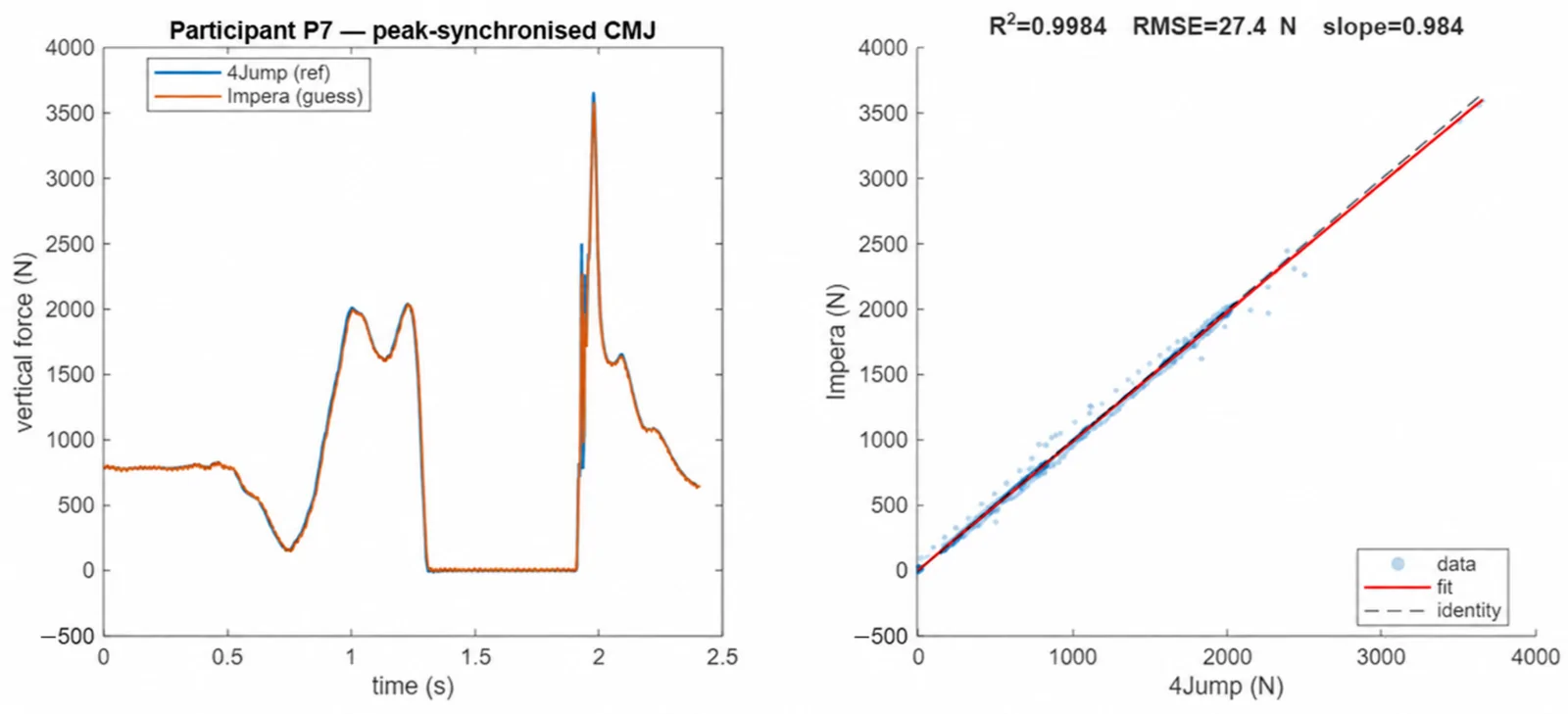
Representative force–time waveform agreement between the Impera and the reference plate (4Jump) for the countermovement jump (participant P7). (**Left**): peak-synchronised vertical force–time signals of the two devices. (**Right**): sample-by-sample scatter with the linear-fit regression line (red) and the identity line (dashed). The coefficient of determination R^2^, slope and the root mean square error (RMSE) are also indicated. The figure refers to the whole-trial analysis; phase-specific agreement for the propulsion and landing braking windows is reported in Appendix A.

For the SJ, the entire vertical force–time waveform recorded by the Impera closely reproduced that of the reference plate in all ten participants, with a near-unity waveform-similarity index, a small (~2%) amplitude underestimation and a negligible offset (Table 6); the residual lag required to align the two signals was ≤2 ms, and the least favourable case (P5) still showed excellent waveform similarity. The same pattern held for the CMJ (Table 7): waveform similarity was again near-unity (R^2^ = 0.997 ± 0.002), with a similar ~2% amplitude underestimation (a_1_ = 0.981 ± 0.006), a negligible offset (a_0_ = 10.360 ± 4.459 N) and a low deviation between signals (RMSD = 29.290 ± 10.979 N), while the residual alignment lag was ≤4 ms (cross-correlation r ≥ 0.99). A representative example of this force–time agreement is shown in Figure 6 for the CMJ (participant P7), in which the reference and Impera waveforms closely match, and the pooled samples lie along the identity line (R^2^ = 0.998; RMSD = 27.4 N; a_1_ = 0.984).

When the analysis was restricted to individual movement phases (Appendix A), device agreement remained high throughout. During the propulsion phase, waveform similarity was near-unity for both tasks (SJ: R^2^ = 0.9938 ± 0.0031, a_1_ = 0.975 ± 0.007, RMSD = 30.970 ± 9.083 N, Table A1; CMJ: R^2^ = 0.9926 ± 0.0030, a_1_ = 0.967 ± 0.008, RMSD = 29.090 ± 8.902 N, Table A2). During the landing braking phase, absolute deviations were larger (SJ: RMSD = 47.260 ± 20.898 N, Table A3; CMJ: RMSD = 73.700 ± 43.702 N, Table A4), consistent with the substantially higher forces generated at impact, yet they corresponded to a similar proportion of the measured signal range than in the propulsion window (1.20% versus 1.84% in the SJ; 1.74% versus 1.72% in the CMJ). Waveform similarity remained high in every participant and phase (R^2^ ≥ 0.970), with amplitude coefficients ranging from 0.933 to 1.005, so that waveform shape was reproduced faithfully throughout. Amplitude was systematically underestimated, and the amplitude coefficient was inversely related to the reference signal range across the whole trial in both tasks (SJ: r = −0.80, *p* = 0.005; CMJ: r = −0.74, *p* = 0.014). Examined by window, this relationship was present in the landing braking phase (SJ: r = −0.84, *p* = 0.002; CMJ: r = −0.75, *p* = 0.012) but not in the propulsion phase of the SJ (r = −0.44, *p* = 0.20) or the braking and propulsion phase of the CMJ (r = 0.35, *p* = 0.32), where the range of forces sampled across participants was narrower. Mean amplitude coefficients did not differ between the two windows (SJ: 0.975 versus 0.975, *p* = 0.98; CMJ: 0.967 versus 0.970, *p* = 0.72).

## 4. Discussion

The present findings show that the Impera platform reproduces known static loads with a small, load-dependent error, achieves between-session variability below 1% under static loading, and reaches good-to-excellent concurrent agreement with the 4Jump reference across nearly all SJ and CMJ variables, an agreement that also held at the level of the complete force–time waveform and within its individual phases.

Both platforms, including the criterion-standard reference, underestimated the lightest tared load by about 4% and progressively approached zero error as the load increased. Because this pattern was common to both devices, which rely on different transducer technologies, strain-gauge and piezoelectric respectively, it is unlikely to reflect a characteristic of either sensing principle. It more plausibly originates upstream, in the reference masses themselves or in the residual zero offset of the two systems, both of which affect the smallest load disproportionately once the error is expressed relative to it. Consistent with this interpretation, the three-way ANOVA attributed most of the variance in measurement error to the applied load ($\eta_{p}^{2}$ = 0.904 for the absolute and 0.946 for the percentage error). A device effect was also detected for the absolute error ($\eta_{p}^{2}$ = 0.254, *p* < 0.001), but its practical magnitude was small: the mean difference between platforms was 0.19 kg (95% CI 0.10 to 0.28), corresponding to 0.9% of the lightest and 0.2% of the heaviest tared load. Notably, no device effect was detected once error was expressed relative to the applied mass ($\eta_{p}^{2}$ = 0.041, *p* = 0.151), so no difference in proportional accuracy was apparent across the tested range. Similar static linearity in a portable load-cell platform and a criterion-standard has been reported previously for other low-cost systems [6], supporting the generalisability of this result beyond the specific device examined in the current study.

A distinctive feature of a platform built on four independent load cells is that its response may vary with where the load is applied. Load position did affect measurement error in the present data ($\eta_{p}^{2}$ = 0.586 and 0.398 for the absolute and percentage error, respectively) and, critically, did not interact with device: no evidence emerged that the two platforms responded differently to load placement. In physical terms the effect was nonetheless modest: the error varied by 0.53 kg across positions in the reference plate and by 0.70 kg in the Impera, and loading at a corner did not differ systematically from loading at the centre. Regional uniformity of the sensing surface has been examined previously for another low-cost platform [6], which likewise reported trivial regional differences. Regional uniformity is a prerequisite for a multi-cell platform, since the summed output should not depend on where the load is applied, and the present data indicate that this holds under static loading across the tested range and positions. The static protocol cannot be extended directly to dynamic tasks, in which the centre of pressure and the distribution of load across the four cells change continuously; the waveform-level analysis addresses that condition, showing close agreement with the reference plate throughout the jump.

Between-session reliability was excellent for both devices, with coefficients of variation below 1% and narrow, near-zero limits of agreement. The reference plate was nonetheless the more repeatable of the two, with a coefficient of variation approximately three-fold smaller and limits of agreement approximately half as wide. This level of repeatability is in line with other validated portable force platforms used in strength and conditioning settings [2,4], and indicates that, once zeroed, a compact load-cell system can provide static measurements that are stable over time, albeit less so than a piezoelectric criterion-standard reference.

Concurrent validity during the jump tasks was good to excellent for essentially every kinetic variable examined (intraclass correlation coefficient from 0.867 to 0.996), following the interpretive thresholds proposed by Koo and Li [10]. The precision of these estimates was, however, uneven. Braking and propulsive force and time were estimated with narrow intervals lying entirely within the excellent range, whereas the variables derived from centre-of-mass acceleration, namely jump height, RSI_MOD_, propulsive impulse and propulsive power/BW, had wider intervals, in some cases extending into the moderate range, so their agreement is estimated with less certainty. This range of agreement is consistent with recent validations of other portable dual-plate systems against Kistler reference plates [2,8] and with earlier work on a one-dimensional portable plate [4]. The Bland–Altman analysis [12] showed a small, consistently positive bias for the propulsive-force variables, indicating a slight underestimation by the Impera platform; a comparable pattern has been attributed, in similarly stacked dual-plate configurations, to minor transmission losses at the plate-to-plate interface used to allow simultaneous recording [2,8]. The limits of agreement varied considerably across variables, from ±2.9% of the mean for propulsive average force in the CMJ to approximately ±14% for jump height and RSI_MOD_ in both tasks, and a single figure would therefore misrepresent the agreement between devices. No threshold for acceptable between-device error has been established for these variables, and the appropriate reference is the variability of the measurement itself. For CMJ variables computed by forward dynamics on a criterion force platform, the between-session minimum difference is approximately 12% of the mean for jump height, 14% for RSI_MOD_ and 8% for propulsive power [27], and between-day coefficients of variation in physically active participants range from about 4% to 11% [25]. The between-device limits of agreement reported here are of the same order or smaller throughout, indicating that between-device error would not exceed the between-session variability of the measurement itself. This holds for jump height derived by the impulse–momentum method, as used here and in the cited studies, but not for flight-time values, which are systematically higher [28].

One interpretive caution applies to the variable-level results. Body mass was derived independently for each device, from its own quiet-standing recording, so that the two processing chains remained separate. A consequence of this choice is that a purely multiplicative amplitude error largely cancels in any variable obtained from centre-of-mass acceleration, since the scaling factor appears in both the numerator and the denominator of (F − BW)/m. Jump height, phase durations, the reactive strength index and body-mass-normalised power are therefore comparatively insensitive to a small amplitude bias, whereas absolute forces and impulses retain it in full. The excellent agreement observed for the former should thus not be read as independent evidence of amplitude accuracy; that property is captured specifically by the amplitude coefficient of the waveform analysis, which is one of the reasons the present study did not rely on discrete variables alone.

Beyond these variable-level results, the linear fit method [13] showed that the entire vertical force–time waveform of the Impera platform closely reproduced that of the reference plate (R^2^ = 0.998 ± 0.001 for the SJ and 0.997 ± 0.002 for the CMJ; RMSD = 25.0 ± 6.0 N and 29.3 ± 10.1 N; a_1_ = 0.982 ± 0.003 and 0.981 ± 0.006), with an approximately 2%, amplitude underestimation and a negligible offset and residual alignment lag. Most previous validations of portable force plates have relied exclusively on discrete outcome variables [2,4,8], leaving open the possibility that summary agreement could coexist with subtler shape distortions of the force–time curve; the present waveform-level confirmation, drawing on a method originally developed for gait analysis and since extended to other biomechanical signals [13,14], closes this gap and supports the use of the Impera platform for applications that depend on the shape of the force–time curve itself, such as phase-specific feedback or the monitoring of changes in force–time profile between conditions or over time.

Partitioning the waveform showed that this agreement was not driven by any single portion of the trial. Waveform similarity remained near-unity during propulsion in both tasks, with amplitude coefficients close to those obtained for the trial as a whole. During the landing braking phase the absolute deviation between devices was considerably larger, which is unsurprising given that the force range sampled at impact was roughly two and a half times that of the propulsive phase. Expressed relative to the range actually sampled, however, that deviation was comparable to the one observed during propulsion, and in the squat jump was smaller. Relative agreement was therefore preserved across the range of forces sampled, but amplitude agreement warrants qualification. The amplitude coefficient decreased as the whole-trial signal range increased in both tasks, indicating greater attenuation in participants generating the highest impact forces. Mean attenuation did not differ between the braking and propulsion window and the landing braking window, however, so the effect is not a simple function of force magnitude; landing strategy is self-selected, and participants who land more stiffly generate peaks that are both higher and faster, so the present design cannot separate the amplitude of the impact from its rate of loading.

The variables reported in Table 4 and Table 5 derive from the braking and propulsive phases of the jump rather than from the landing. This reflects standard practice: in the CMJ and SJ the variables used for athlete monitoring are computed up to take-off, whereas in tasks such as the drop jump the ground contact that follows impact is itself part of the measurement [28]. No relationship between the amplitude coefficient and the sampled force range was detectable over the corresponding analysis windows, so this attenuation does not affect the reported concurrent-agreement estimates. Phase boundaries were taken from the reference signal for both devices, so this analysis compares waveform agreement within corresponding phases rather than the ability of the Impera to detect those boundaries; the latter is reflected in the agreement observed for braking and propulsive time, which were derived independently from each device (Table 4). Phase-specific values are reported in Appendix A.

This three-level validation, accuracy against known references, between-session reliability, and concurrent agreement at both the variable and the waveform level, follows the same approach previously used by our group to validate other field-based instruments, including a portable GNSS receiver for running [20,29], an isometric bench for knee assessment [21], and a smartphone-based jump-height application [22]. The consistency of the present results with this body of work reinforces construct and concurrent validity as the appropriate framework for evaluating new test platform sport-science tools before their adoption in research or applied practice.

From a practical standpoint, the combination of affordability, portability, and the accuracy, reliability and validity documented here makes the Impera platform a viable option for strength and conditioning practitioners, coaches and researchers who lack routine access to criterion-standard, tared instrumentation, in line with the broader shift toward accessible monitoring technology in sport science [3,5].

This study has some limitations. The dynamic protocol involved ten physically active men performing unloaded squat and countermovement jumps only; the present results may not extend to loaded jumps, to other populations such as female, youth or clinical samples, or to more demanding tasks such as the drop jump, which places a greater mechanical and sampling-rate burden on portable, unanchored platforms [2]. The dynamic sample was also small, and the width of the confidence intervals around the lower intraclass correlation coefficients reflects this: precision was high where agreement was excellent but limited for the two variables showing good rather than excellent agreement. The multiple samples contributing to each waveform-level fit are serially dependent and characterise the fidelity of the individual waveform; they do not increase the number of independent observations available for estimating between-participant agreement. Larger samples would be required to estimate agreement with comparable precision across all variables. Between-session reliability was assessed under static loading only, and the test–retest reliability of the platform during jumping remains to be established. Since biological variability between jumps compounds instrumental variability, dynamic reliability cannot be inferred from the static results. The reference masses were determined with a class III mechanical column scale bearing CE M certification (Max 150 kg, e = 100 g), for which the manufacturer specifies a factory accuracy of ±100 g. Because the discs were weighed individually, the worst-case uncertainty on the cumulative loads ranges from 0.20 kg at the lightest to 0.60 kg at the heaviest, that is below 1% throughout. This accounts for part, but not all, of the 4% deviation observed at the lightest load, where both platforms underestimated to a similar extent; the remainder is more likely attributable to the residual zero offset of the two platforms, which affects the smallest load disproportionately when the error is expressed in relative terms. The manufacturer does not declare a natural frequency for the Impera load cells or for the assembled platform, whereas the reference plate is specified at approximately 150 Hz. Since it is the structural response of the assembled system, rather than that of the individual sensing element, that governs the fidelity of impact transients, a natural frequency lower than that of the reference plate would be the most plausible mechanistic explanation for the small amplitude attenuation observed at the highest forces; the absence of this specification is in itself a reason for caution in extrapolating the present findings to drop jumps and other high-impact tasks. Landing technique was not standardised or quantified. Since landing strategy determines both the magnitude and the rate of loading of the impact peak, the observed relationship between amplitude attenuation and impact force may reflect either the force itself or the speed at which it is applied, and the present design cannot distinguish between them. Because the 1000 Hz output is obtained by averaging ten consecutive samples, it involves a mild low-pass action; over the frequency range relevant to vertical jumping this attenuates amplitude by less than 2% and cannot account for the underestimation observed at the highest forces. Only a single unit of the Impera platform was tested, and the stacked dual-plate configuration used to enable simultaneous recording, while standard in this line of research [2,15], was in this case also dictated by geometry, the reference plate being the larger of the two devices. This arrangement may itself introduce a small interface-related bias distinct from the platform’s intrinsic measurement error, and it places the Impera platform on a rigid metal structure rather than on a floor. The reference plate is at least as stiff as a typical sports surface, so the arrangement is unlikely to have introduced compliance, but the transfer of these findings to platforms resting directly on floors of differing stiffness remains to be established.

A further consideration concerns the waveform analysis: the two signals were temporally aligned on the peak force of the landing phase, which falls within one of the analysis windows. Agreement within the landing window may therefore be marginally favoured by the synchronisation procedure itself, whereas the propulsion window, which lies away from the alignment point, provides the more conservative estimate.

Future studies should extend this validation to loaded and higher-impact tasks, to larger and more diverse samples, and to outcomes beyond the vertical component of the ground reaction force.

## 5. Conclusions

The Impera portable force platform showed a small, load-dependent measurement error of a magnitude similar to that of the criterion-standard reference, excellent between-session reliability under static loading (coefficient of variation below 1%), and good-to-excellent concurrent validity with the reference plate across nearly all SJ and CMJ variables, confirmed at the level of the entire force–time waveform (R^2^ ≥ 0.997) and within its propulsion and landing phases. The evidence therefore covers accuracy and between-session repeatability under static loading, and concurrent agreement at both the variable and the waveform level during vertical jumping; test–retest reliability during jumping was not assessed, and interchangeability with criterion-standard instrumentation was not formally tested. These findings support the use of the Impera platform as an accurate and reliable alternative to criterion-standard instrumentation for static loading assessment, and as valid for vertical jump testing, in field and applied sport-science settings. Its portability and affordability, combined with the validation evidence reported here, make it particularly suitable for strength and conditioning practitioners and researchers without routine access to laboratory-grade equipment. Future research should extend this validation to loaded and more demanding tasks such as the drop jump, to other populations including female and youth athletes, and to biomechanical outcomes beyond the vertical component of the ground reaction force.

## Figures and Tables

**Table 1:** Percentage error (mean and standard deviation, %) of each platform at the five tared loads.

Load (kg)	4Jump	Impera
21.6	−4.084 (0.289)	−3.745 (1.163)
41.9	−1.112 (0.432)	−1.022 (0.948)
62.5	−0.484 (0.291)	−0.770 (0.730)
82.3	0.744 (0.324)	0.384 (0.483)
103.2	0.629 (0.340)	0.057 (0.429)

Note: all data are expressed as mean (SD).

**Table 2:** Three-way ANOVA (fixed factors: load, device and plate location; Type III sums of squares) for the absolute and percentage measurement error.

Effect	*df*	Absolute Error (kg)			Percentage Error (%)		
		*F*	*p*	$\eta_{p}^{2}$	*F*	*p*	$\eta_{p}^{2}$
Load	4, 50	117.585	<0.001	0.904	218.792	<0.001	0.946
Device	1, 50	17.059	<0.001	0.254	2.124	0.151	0.041
Position	4, 50	17.703	<0.001	0.586	8.273	<0.001	0.398
Load × Device	4, 50	6.846	<0.001	0.354	2.286	0.073	0.155
Device × Position	4, 50	1.360	0.261	0.098	0.463	0.763	0.036
Load × Position	16, 50	1.904	0.043	0.379	0.929	0.543	0.229
Load × Device × Position	16, 50	1.212	0.292	0.279	0.985	0.487	0.240

Note: *df*, degrees of freedom (numerator, denominator), identical for both error models. $\eta_{p}^{2}$, partial eta squared. Eta-squared values (η^2^, proportion of total variance) are reported in Table S1.

**Table 3:** Between-session test–retest reliability of each platform: coefficient of variation (CV%) and Bland–Altman bias and 95% limits of agreement (LoA) of the session-averaged measures, with 95% confidence interval for CV% and bias.

Device	CV% [95% CI]	Between-Session Bias, kg [95% CI]	95% LoA (kg)
4Jump (Reference)	0.155 [0.110, 0.199]	−0.003 [−0.076, 0.070]	−0.349 to 0.343
Impera (New device)	0.530 [0.297, 0.762]	0.007 [−0.174, 0.188]	−0.852 to 0.865

**Table 4:** Concurrent agreement [ICC_2,1_ with 95% CI] between the Impera and the reference force plate for the CMJ and SJ.

Variable	CMJ—ICC_2,1_ [95% CI]	SJ—ICC_2,1_ [95% CI]
Jump height [m]	0.899 [0.666, 0.974] ^a^	0.885 [0.625, 0.970] ^a^
RSI_MOD_ [a.u.]	0.924 [0.739, 0.980]	0.952 [0.826, 0.988]
Braking average force [N]	0.996 [0.986, 0.999]	—
Braking impulse [N·s]	0.990 [0.964, 0.998]	—
Braking time [s]	0.982 [0.933, 0.995]	—
Propulsive average force [N]	0.995 [0.980, 0.999]	0.976 [0.911, 0.994]
Propulsive impulse [N·s]	0.867 [0.577, 0.965] ^a^	0.871 [0.585, 0.966] ^a^
Propulsive time [s]	0.992 [0.969, 0.998]	0.991 [0.967, 0.998]
Mean propulsive power/BW [W·kg^−1^]	0.958 [0.850, 0.989]	0.985 [0.943, 0.996]

Note: ICC_2,1_ computed following Shrout and Fleiss [9]; *n* = 10, two concurrent measurements (Impera and reference plate). Classification refers to the point estimate; for several variables the confidence interval spans more than one interpretive category, and the interval rather than the point estimate should guide interpretation. —, not applicable (the SJ has no braking phase); CI, confidence interval; RSI_MOD_, modified reactive strength index. ^a^ Point estimate in the good range.

**Table 5:** Bland–Altman agreement between the Impera and the reference force plate for the CMJ and the SJ: descriptive mean, bias and 95% limits of agreement, in measurement units and as a percentage of the mean.

Variable	Mean	Bias	95% LoA	LoA (%)
Jump height_CMJ_ [m]	0.353	0.004	−0.045 to 0.053	±13.9
Jump height_SJ_ [m]	0.286	−0.007	−0.047 to 0.033	±14.0
RSI_MOD CMJ_ [a.u.]	0.792	0.007	−0.100 to 0.115	±13.6
RSI_MOD SJ_ [a.u.]	0.932	−0.029	−0.162 to 0.104	±14.3
Braking average force_CMJ_ [N]	567.3	−3.073	−33.32 to 27.18	±5.3
Braking impulse_CMJ_ [N·s]	99.3	0.688	−8.12 to 9.49	±8.9
Braking time_CMJ_ [s]	0.177	0.002	−0.009 to 0.014	±6.5
Propulsive average force_CMJ_ [N]	999.6	1.170	−27.99 to 30.33	±2.9
Propulsive average force_SJ_ [N]	958.5	7.833	−69.08 to 84.75	±8.0
Propulsive impulse_CMJ_ [N·s]	268.8	0.886	−17.51 to 19.28	±6.8
Propulsive impulse_SJ_ [N·s]	295.7	4.084	−25.53 to 33.70	±10.0
Propulsive time_CMJ_ [s]	0.274	0.001	−0.010 to 0.012	±4.0
Propulsive time_SJ_ [s]	0.274	0.002	−0.008 to 0.012	±3.7
Mean propulsive power/BW_CMJ_ [W·kg^−1^]	14.48	0.088	−1.249 to 1.425	±9.2
Mean propulsive power/BW_SJ_ [W·kg^−1^]	12.74	−0.093	−1.153 to 0.968	±8.3

Note. *n* = 10. Mean is the average of the two devices. Bias is the mean difference (4Jump minus Impera); positive values indicate underestimation by the Impera. LoA (%) is the half-width of the limits of agreement expressed as a percentage of the mean. The three braking variables apply to the CMJ only, as the SJ has no braking phase. RSI_MOD_, modified reactive strength index; BW, body weight.

**Table 6:** Force–time waveform agreement for the squat jump (linear fit method): reference and Impera signal ranges, root-mean-square deviation (RMSD), waveform-similarity index (R^2^), amplitude coefficient (a_1_) and offset (a_0_).

Participant	Ref Signal Range (N)	Impera Signal Range (N)	RMSD (N)	R^2^	a_1_	a_0_ (N)
P1	3820	3667	24.1	0.999	0.983	11.2
P2	3353	3246	19.7	0.999	0.987	5.7
P3	3764	3684	26.7	0.998	0.981	8.6
P4	1448	1391	19.3	0.997	0.986	6.1
P5	5040	4856	39.3	0.997	0.982	9.8
P6	5477	5397	28.9	0.998	0.979	3.6
P7	4101	4002	26.1	0.999	0.984	8.1
P8	3600	3491	19.0	0.999	0.982	8.5
P9	5558	5298	22.8	0.999	0.981	12.1
P10	6113	5809	24.5	0.999	0.979	14.7
Mean ± SD	—	—	25.0 ± 6.0	0.998 ± 0.001	0.982 ± 0.003	8.8 ± 3.3

Note: a_1_, amplitude coefficient (regression slope); a_0_, offset (regression intercept). Signal range = maximum − minimum force, reported per participant for transparency and not pooled, as amplitude agreement between devices is quantified by a_1_. All temporal lags were ≤2 ms and cross-correlation r ≥ 0.99.

**Table 7:** Force–time waveform agreement for the countermovement jump (linear fit method): reference and Impera signal ranges, root-mean-square deviation (RMSD), waveform-similarity index (R^2^), amplitude coefficient (a_1_) and offset (a_0_).

Participant	Ref Signal Range (N)	Impera Signal Range (N)	RMSD (N)	R^2^	a_1_	a_0_ (N)
P1	5104	4806	56.7	0.992	0.969	18.8
P2	2840	2737	22.1	0.999	0.987	5.2
P3	4600	4485	27.8	0.998	0.981	9.0
P4	1723	1659	16.5	0.998	0.987	6.5
P5	5868	5575	37.1	0.997	0.972	17.6
P6	5904	5847	23.2	0.999	0.978	8.4
P7	3663	3601	27.4	0.998	0.984	10.3
P8	3528	3437	27.9	0.998	0.980	11.0
P9	5167	5106	25.9	0.998	0.983	8.4
P10	3578	3488	28.3	0.998	0.985	8.4
Mean ± SD	—	—	29.3 ± 10.1	0.997 ± 0.002	0.981 ± 0.006	10.4 ± 4.5

Note: a_1_, amplitude coefficient (regression slope); a_0_, offset (regression intercept). Signal range = maximum − minimum force, reported per participant for transparency and not pooled, as amplitude agreement between devices is quantified by a_1_. All temporal lags were ≤4 ms and cross-correlation r ≥ 0.99.

## Data Availability

The raw data and the code supporting the conclusions of this article will be made available by the authors on request.

## Supplementary Materials

The following supporting information can be downloaded at: https://www.mdpi.com/article/10.3390/s26185850/s1, Table S1: Eta-squared (η2) effect sizes for the three-way mixed-effects ANOVAs on the absolute and percentage measurement error; Figure S1: Bland–Altman plot for jump height (CMJ); Figure S2: Bland–Altman plot for RSI_MOD (CMJ); Figure S3: Bland–Altman plot for braking average force (CMJ); Figure S4: Bland–Altman plot for braking impulse (CMJ); Figure S5: Bland–Altman plot for braking time (CMJ); Figure S6: Bland–Altman plot for propulsive average force (CMJ); Figure S7: Bland–Altman plot for propulsive impulse (CMJ); Figure S8: Bland–Altman plot for propulsive time (CMJ); Figure S9: Bland–Altman plot for mean propulsive power relative to body mass (CMJ); Figure S10: Bland–Altman plot for jump height (SJ); Figure S11: Bland–Altman plot for RSI_MOD (SJ); Figure S12: Bland–Altman plot for propulsive average force (SJ); Figure S13: Bland–Altman plot for propulsive impulse (SJ); Figure S14: Bland–Altman plot for propulsive time (SJ); Figure S15: Bland–Altman plot for mean propulsive power relative to body mass (SJ).

## Abbreviations

The following abbreviations are used in this manuscript:

CI	Confidence intervals
CMJ	Countermovement jump
CV	Coefficient of variation
FS	Full scale
FSO	Full scale output
ICC	Intraclass correlation coefficient
JH	Jump height
LoA	Limits of agreement
RMSD	Root mean square deviation
SD	Standard deviation
SJ	Squat jump

## Appendix A

**Table A1:** Force–time waveform agreement for the squat jump during the propulsion phase (movement onset to take-off; linear fit method): reference and Impera signal ranges, root-mean-square deviation (RMSD), waveform-similarity index (R^2^), amplitude coefficient (a_1_) and offset (a_0_).

Participant	Ref Signal Range (N)	Impera Signal Range (N)	RMSD (N)	R^2^	a_1_	a_0_ (N)
P1	1773	1737	36.8	0.993	0.972	36.3
P2	1740	1706	36	0.993	0.972	30.3
P3	1621	1598	27.4	0.995	0.985	12.8
P4	1074	1075	24.9	0.988	0.973	24.1
P5	1849	1807	26.9	0.997	0.973	34.2
P6	1690	1646	36.3	0.992	0.976	17.7
P7	1997	1964	50.2	0.990	0.965	46.1
P8	1628	1612	23.5	0.996	0.986	11
P9	1456	1444	18.3	0.997	0.981	17.4
P10	2007	1909	29.4	0.996	0.968	44.6
Mean ± SD	—	—	31.0 ± 9.1	0.994 ± 0.003	0.975 ± 0.007	27.4 ± 12.8

Note: a_1_, amplitude coefficient (regression slope); a_0_, offset (regression intercept). Signal range = maximum − minimum force within the propulsion window, reported per participant for transparency and not pooled, as amplitude agreement between devices is quantified by a_1_. All temporal lags were ≤2 ms.

**Table A2:** Force–time waveform agreement for the countermovement jump during the braking and propulsion phase (onset of braking to take-off; linear fit method): reference and Impera signal ranges, root-mean-square deviation (RMSD), waveform-similarity index (R^2^), amplitude coefficient (a_1_) and offset (a_0_).

Participant	Ref Signal Range (N)	Impera Signal Range (N)	RMSD (N)	R^2^	a_1_	a_0_ (N)
P1	1856	1802	42.4	0.989	0.961	49
P2	1792	1726	37.8	0.987	0.959	49
P3	1389	1354	22	0.994	0.972	23.5
P4	1180	1124	21.9	0.991	0.952	43.6
P5	1719	1675	30.1	0.994	0.965	43
P6	1694	1644	26	0.994	0.964	33.1
P7	2019	1963	41.2	0.990	0.97	38.7
P8	1893	1875	30.7	0.996	0.979	21.7
P9	1570	1545	17.1	0.996	0.975	22
P10	1630	1562	21.7	0.994	0.972	33.7
Mean ± SD	—	—	29.1 ± 8.9	0.993 ± 0.003	0.967 ± 0.008	35.7 ± 10.7

Note: a_1_, amplitude coefficient (regression slope); a_0_, offset (regression intercept). Signal range = maximum − minimum force within the braking–propulsion window, reported per participant for transparency and not pooled, as amplitude agreement between devices is quantified by a_1_. All temporal lags were ≤4 ms.

**Table A3:** Force–time waveform agreement for the squat jump during the landing braking phase (touchdown to the return of vertical force to body weight; linear fit method): reference and Impera signal ranges, root-mean-square deviation (RMSD), waveform-similarity index (R^2^), amplitude coefficient (a_1_) and offset (a_0_).

Participant	Ref Signal Range (N)	Impera Signal Range (N)	RMSD (N)	R^2^	a_1_	a_0_ (N)
P1	3749	3573	39.1	0.997	0.974	22.5
P2	3238	3114	23.3	0.999	0.982	14.4
P3	3693	3558	56.1	0.995	0.971	19.1
P4	1368	1321	28.6	0.982	0.989	0.4
P5	4972	4735	93.3	0.991	0.973	18.2
P6	5405	5259	64.1	0.996	0.964	28.8
P7	4035	3898	33.3	0.998	0.983	8.0
P8	3541	3423	32.1	0.998	0.977	9.4
P9	5429	5136	50.3	0.997	0.973	24.6
P10	6032	5698	52.4	0.998	0.966	40.2
Mean ± SD	—	—	47.3 ± 20.9	0.995 ± 0.005	0.975 ± 0.008	18.6 ± 11.4

Note: a_1_, amplitude coefficient (regression slope); a_0_, offset (regression intercept). Signal range = maximum − minimum force within the landing window, and includes the impact peak; RMSD values are therefore larger in absolute terms than in the propulsion window, while representing an equal or smaller proportion of the measured range (mean 1.20%). Ranges are reported per participant for transparency and not pooled, as amplitude agreement between devices is quantified by a_1_. All temporal lags were ≤2 ms.

**Table A4:** Force–time waveform agreement for the countermovement jump during the landing braking phase (touchdown to the return of vertical force to body weight; linear fit method): reference and Impera signal ranges, root-mean-square deviation (RMSD), waveform-similarity index (R^2^), amplitude coefficient (a_1_) and offset (a_0_).

Participant	Ref Signal Range (N)	Impera Signal Range (N)	RMSD (N)	R^2^	a_1_	a_0_ (N)
P1	5041	4771	165.8	0.970	0.933	82.3
P2	2736	2624	21.8	0.997	0.984	8.3
P3	4511	4409	92.8	0.993	0.973	18.8
P4	1656	1608	23.6	0.990	1.005	-12.8
P5	5787	5496	113.6	0.992	0.947	64
P6	5735	5621	66	0.998	0.972	11.2
P7	3422	3371	42.9	0.995	0.976	21.3
P8	3473	3365	56.1	0.995	0.965	28.4
P9	5057	4935	67.1	0.994	0.968	35.2
P10	3497	3271	87.3	0.990	0.973	27.6
Mean ± SD	—	—	73.7 ± 43.7	0.991 ± 0.008	0.970 ± 0.019	28.4 ± 27.4

Note: a_1_, amplitude coefficient (regression slope); a_0_, offset (regression intercept). Signal range = maximum − minimum force within the landing window, and includes the impact peak; RMSD values are therefore larger in absolute terms than in the braking–propulsion window, while representing essentially the same proportion of the measured range (mean 1.74% versus 1.72%). Ranges are reported per participant for transparency and not pooled, as amplitude agreement between devices is quantified by a_1_. All temporal lags were ≤4 ms.

## Appendix B

**Table A5:** Percentage error by load magnitude and load position—reference plate (4Jump).

Load (kg)	Centre	Front-Left	Front-Right	Rear-Left	Rear-Right	Mean
21.6	−4.051	−4.435	−3.987	−4.193	−3.755	−4.084
41.9	−1.105	−1.660	−1.104	−1.226	−0.466	−1.112
62.5	−0.266	−0.917	−0.486	−0.569	−0.180	−0.484
82.3	0.863	0.398	0.760	0.483	1.217	0.744
103.2	0.619	0.238	0.736	0.455	1.097	0.629
Mean ± SD	−0.788 ± 1.874	−1.275 ± 1.853	−0.816 ± 1.840	−1.010 ± 1.812	−0.417 ± 1.902	−0.861 ± 1.802

Note: Values are percentage errors relative to the known masses, averaged across the two sessions. Column SDs reflect the variation of error across load magnitudes rather than variability between repeated measurements at the same load. Positions refer to the centre and the four corners of the plate surface.

**Table A6:** Percentage error by load magnitude and load position—Impera.

Load (kg)	Centre	Front-Left	Front-Right	Rear-Left	Rear-Right	Mean
21.6	−4.279	−3.418	−3.786	−4.509	−2.733	−3.745
41.9	−1.162	−2.055	0.029	−1.006	−0.918	−1.022
62.5	−0.983	−1.759	−0.993	−0.242	0.127	−0.770
82.3	0.467	0.253	−0.034	0.434	0.798	0.384
103.2	0.031	−0.498	−0.116	0.180	0.687	0.057
Mean ± SD	−1.185 ± 1.805	−1.495 ± 1.488	−0.980 ± 1.578	−1.029 ± 2.097	−0.408 ± 1.392	−1.019 ± 1.660

Note: Values are percentage errors relative to the known masses, averaged across the two sessions. Column SDs reflect the variation of error across load magnitudes rather than variability between repeated measurements at the same load. Positions refer to the centre and the four corners of the plate surface.
