# Supplementary material for: Accuracy, Reliability and Waveform-Level Agreement Between the Impera Force Platform and a Criterion-Standard Force Plate
Source: Sensors (Basel). 2026 Sep 15;26(18):5850. doi: 10.3390/s26185850 (PMC13611723; doi:10.3390/s26185850)
Supplement: Supplementary file 1 [file sensors-26-05850-s001.zip › sensors-4500143-supplementary.pdf]

Supplementary Materials

Validation of a portable force platform (Impera) against a criterion-standard force plate (4Jump) during static loading and vertical jumping.

Table S1. Eta-squared (η^2) effect sizes for the three-way mixed-effects ANOVAs on the absolute and percentage measurement error.

Effect	η^2 absolute error (kg)	η^2 percentage error (%)
Load	0.681	0.873
Device	0.025	0.002
Position	0.102	0.033
Load × Device	0.040	0.009
Device × Position	0.008	0.002
Load × Position	0.044	0.015
Load × Device × Position	0.028	0.016

Note. η^2 was computed as the ratio of the effect sum of squares to the total sum of squares, and therefore expresses the proportion of the total variance in measurement error attributable to each factor. Partial eta-squared values (η^2_P), together with the corresponding F and p values and degrees of freedom, are reported in Table 2 of the main manuscript.

Figures S1–S15 show the Bland–Altman analysis for every jump variable reported in Table 5. In each plot the difference between devices (4Jump minus Impera) is plotted against the mean of the two devices. The solid line indicates the bias (mean difference) and the dashed lines the 95% limits of agreement (bias ± 1.96 SD). Figures S1–S9 refer to the countermovement jump (CMJ) and Figures S10–S15 to the squat jump (SJ); the SJ has no braking phase, so braking variables are not reported for that task.

Descriptive Statistics Countermovement jump (CMJ)

Descriptive Statistics

	Mean (arithmetic)	95% Confidence Interval Mean		Std. Deviation
		Lower	Upper	
jump height (m) [4jump]	0.355	0.322	0.388	0.046
jump height (m) [Impera]	0.351	0.308	0.394	0.060
RSI (AU) [4jump]	0.796	0.701	0.890	0.132
RSI (AU) [Impera]	0.788	0.690	0.887	0.138
braking average force (N) [4jump]	565.760	441.052	690.468	174.330
braking average force (N) [Impera]	568.833	444.610	693.056	173.651
braking impulse (N·s) [4jump]	99.612	77.296	121.929	31.197
braking impulse (N·s) [Impera]	98.925	76.794	121.055	30.936
braking time (s) [4jump]	0.178	0.155	0.201	0.032
braking time (s) [Impera]	0.176	0.154	0.198	0.031
propulsive average force (N) [4jump]	1000.161	901.451	1098.871	137.987
propulsive average force (N) [Impera]	998.992	899.739	1098.244	138.746
propulsive impulse (N·s) [4jump]	269.239	258.664	279.815	14.783
propulsive impulse (N·s) [Impera]	268.353	254.241	282.466	19.728
propulsive time (s) [4jump]	0.274	0.245	0.303	0.041
propulsive time (s) [Impera]	0.273	0.243	0.304	0.042
power relative to body mass (watt·kg^{-1}) [4jump]	14.528	12.950	16.105	2.205
power relative to body mass (watt·kg^{-1}) [Impera]	14.440	12.791	16.089	2.305

Intraclass Correlation Countermovement jump (CMJ)

***Intraclass Correlation* jump height (CMJ).**

Type	Point Estimate	Lower 95% CI	Upper 95% CI
ICC2,1	0.899	0.666	0.974

Note. 10 subjects and 2 raters/measurements. ICC type as referenced by Shrout & Fleiss (1979).

***Intraclass Correlation* RSI$_{MOD}$ (CMJ).**

Type	Point Estimate	Lower 95% CI	Upper 95% CI
ICC2,1	0.924	0.739	0.980

Note. 10 subjects and 2 raters/measurements. ICC type as referenced by Shrout & Fleiss (1979).

***Intraclass Correlation* braking average force (CMJ).**

Type	Point Estimate	Lower 95% CI	Upper 95% CI
ICC2,1	0.996	0.986	0.999

Note. 10 subjects and 2 raters/measurements. ICC type as referenced by Shrout & Fleiss (1979).

***Intraclass Correlation* braking impulse (CMJ).**

Type	Point Estimate	Lower 95% CI	Upper 95% CI
ICC2,1	0.990	0.964	0.998

Note. 10 subjects and 2 raters/measurements. ICC type as referenced by Shrout & Fleiss (1979).

***Intraclass Correlation* braking time (CMJ).**

Type	Point Estimate	Lower 95% CI	Upper 95% CI
ICC2,1	0.982	0.933	0.995

Note. 10 subjects and 2 raters/measurements. ICC type as referenced by Shrout & Fleiss (1979).

***Intraclass Correlation* propulsive average force (CMJ).**

Type	Point Estimate	Lower 95% CI	Upper 95% CI
ICC2,1	0.995	0.980	0.999

Note. 10 subjects and 2 raters/measurements. ICC type as referenced by Shrout & Fleiss (1979).

***Intraclass Correlation* propulsive impulse (CMJ).**

Type	Point Estimate	Lower 95% CI	Upper 95% CI
ICC2,1	0.867	0.577	0.965

Note. 10 subjects and 2 raters/measurements. ICC type as referenced by Shrout & Fleiss (1979).

***Intraclass Correlation* propulsive time (CMJ).**

Type	Point Estimate	Lower 95% CI	Upper 95% CI
ICC2,1	0.992	0.969	0.998

Note. 10 subjects and 2 raters/measurements. ICC type as referenced by Shrout & Fleiss (1979).

***Intraclass Correlation* power relative to body mass (CMJ).**

Type	Point Estimate	Lower 95% CI	Upper 95% CI
ICC2,1	0.958	0.850	0.989

Note. 10 subjects and 2 raters/measurements. ICC type as referenced by Shrout & Fleiss (1979).

Bland-Altman Plots Countermovement jump (CMJ)

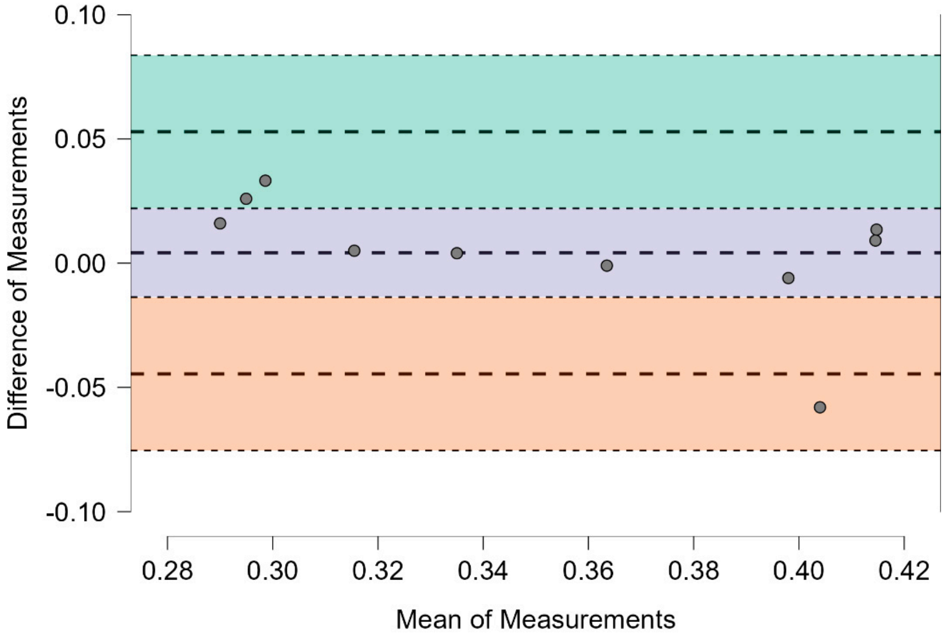

Figure S1. Bland–Altman plot for jump height (CMJ).

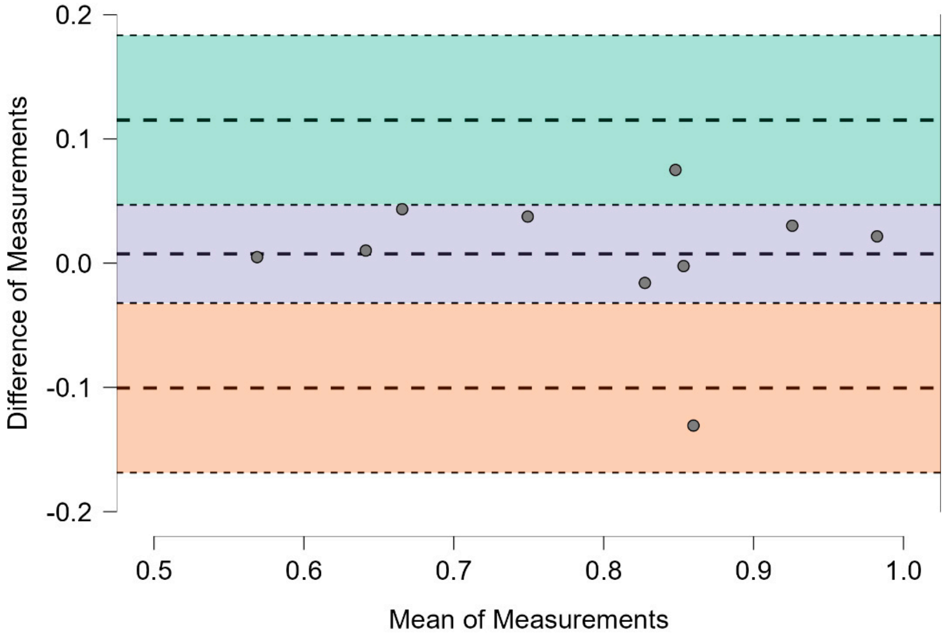

Figure S2. Bland–Altman plot for RSI_{MOD} (CMJ).

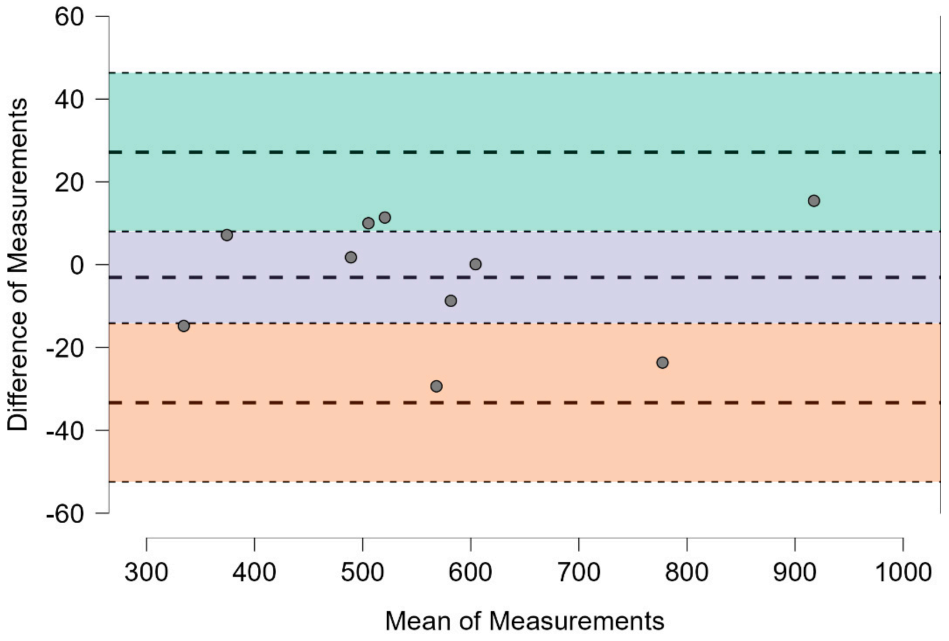

Figure S3. Bland–Altman plot for braking average force (CMJ).

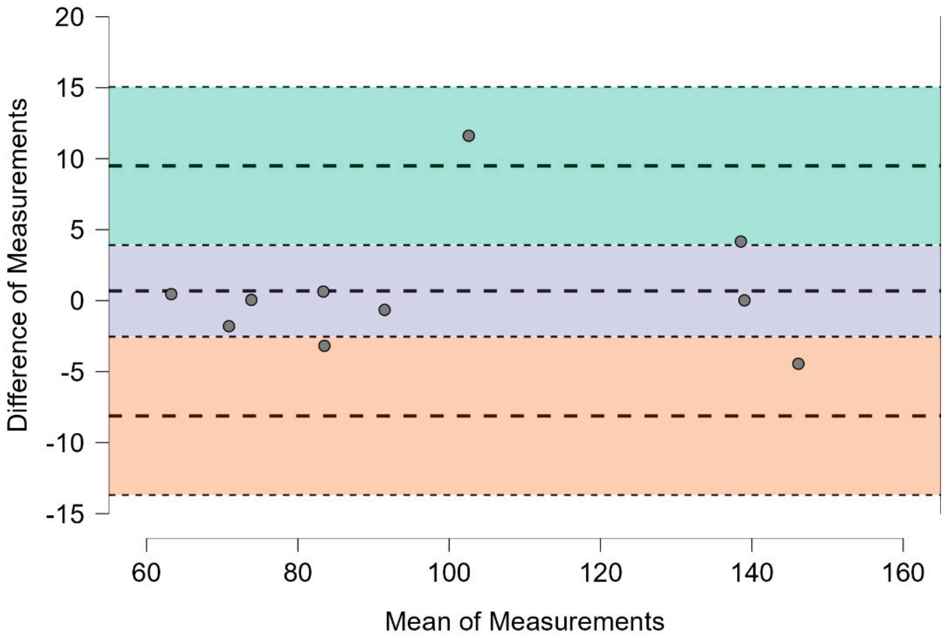

Figure S4. Bland–Altman plot for braking impulse (CMJ).

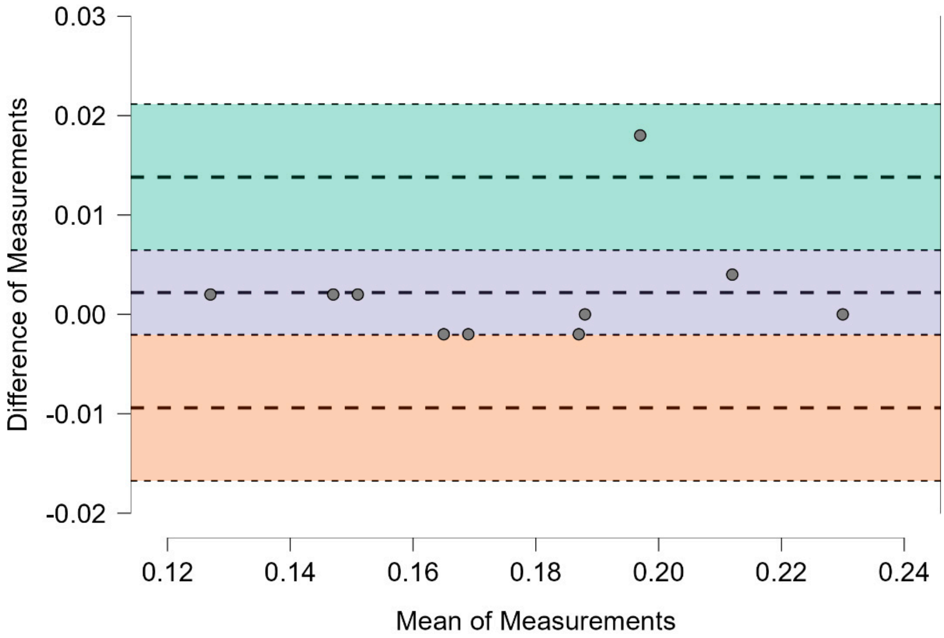

Figure S5. Bland–Altman plot for braking time (CMJ).

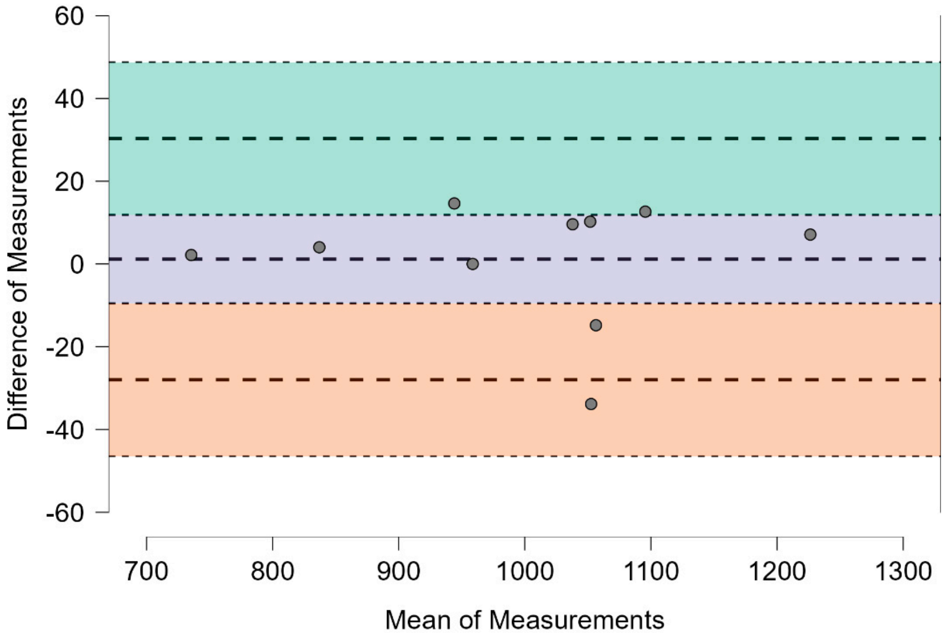

Figure S6. Bland–Altman plot for propulsive average force (CMJ).

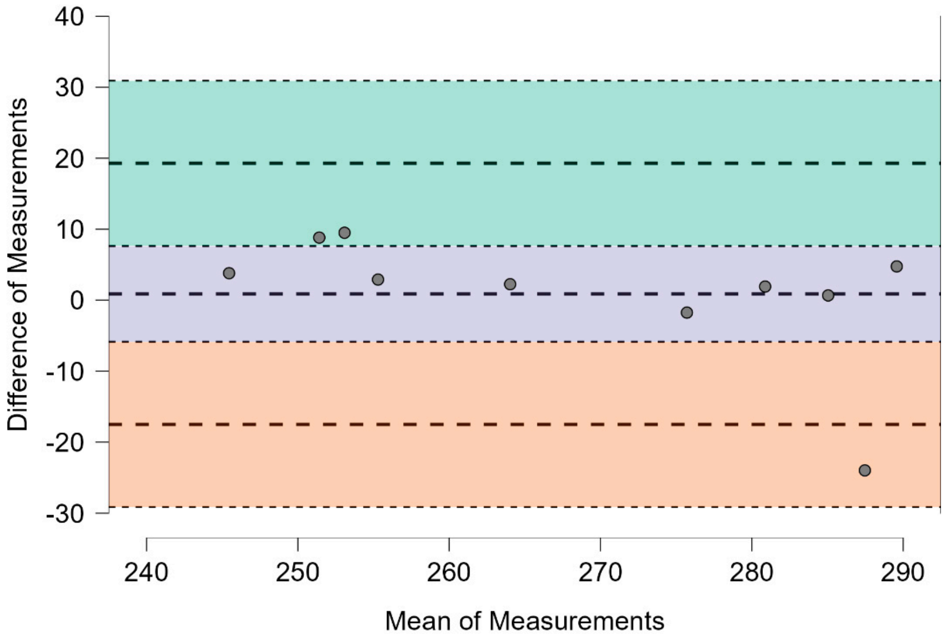

Figure S7. Bland–Altman plot for propulsive impulse (CMJ).

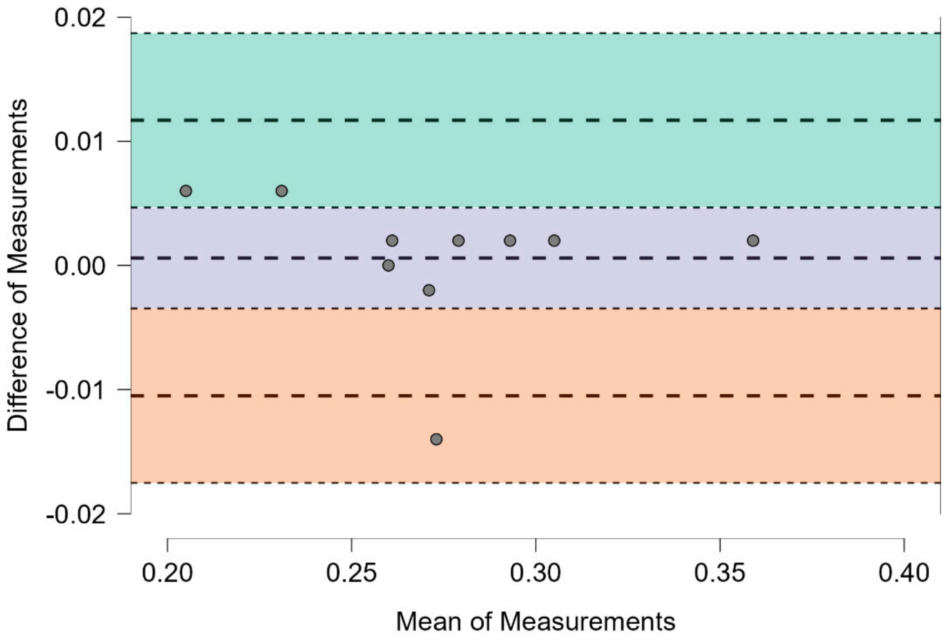

Figure S8. Bland–Altman plot for propulsive time (CMJ).

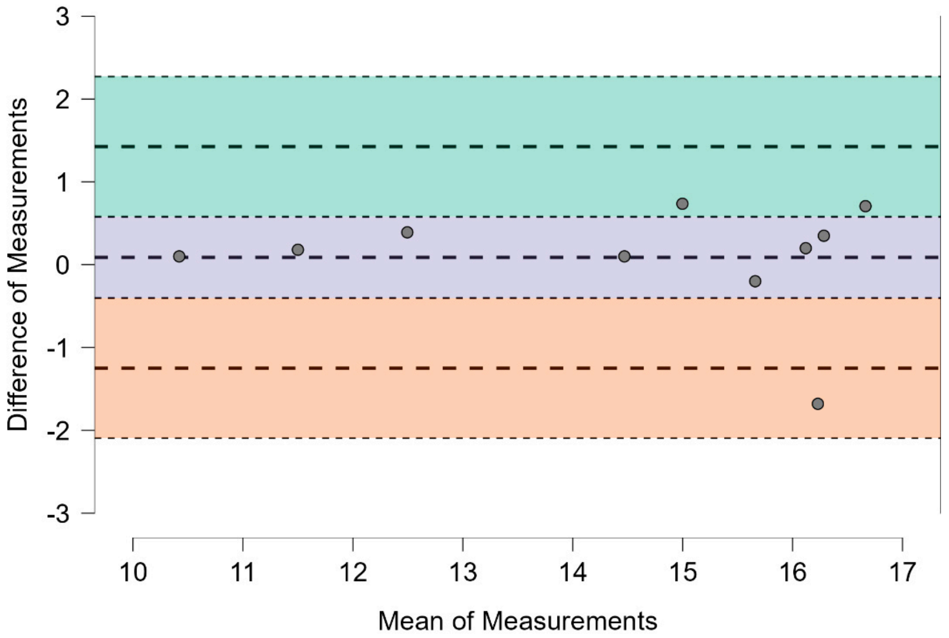

Figure S9. Bland–Altman plot for mean propulsive power relative to body mass (CMJ).

Bland-Altman Tables Countermovement jump (CMJ)

jump height (m) [4jump] - jump height (m) [Impera]

Bias & Limits	Point Value	Lower 95% CI	Upper 95% CI
Mean difference + 1.96 SD	0.053	0.022	0.084
Mean difference	0.004	-0.014	0.022
Mean difference - 1.96 SD	-0.045	-0.075	-0.014

RSI_{MOD} (AU) [4jump] - RSI_{MOD} (AU) [Impera]

Bias & Limits	Point Value	Lower 95% CI	Upper 95% CI
Mean difference + 1.96 SD	0.115	0.047	0.183
Mean difference	0.007	-0.032	0.047
Mean difference - 1.96 SD	-0.100	-0.169	-0.032

braking average force (N) [4jump] - braking average force (N) [Impera]

Bias & Limits	Point Value	Lower 95% CI	Upper 95% CI
Mean difference + 1.96 SD	27.178	8.055	46.301
Mean difference	-3.073	-14.113	7.968
Mean difference - 1.96 SD	-33.323	-52.446	-14.200

braking impulse (N·s) [4jump] - braking impulse (N·s) [Impera]

Bias & Limits	Point Value	Lower 95% CI	Upper 95% CI
Mean difference + 1.96 SD	9.491	3.926	15.057
Mean difference	0.688	-2.525	3.901
Mean difference - 1.96 SD	-8.116	-13.681	-2.551

braking time (s) [4jump] - braking time (s) [Impera]

Bias & Limits	Point Value	Lower 95% CI	Upper 95% CI
Mean difference + 1.96 SD	0.014	0.006	0.021
Mean difference	0.002	-0.002	0.006
Mean difference - 1.96 SD	-0.009	-0.017	-0.002

propulsive average force (N) [4jump] - propulsive average force (N) [Impera]

Bias & Limits	Point Value	Lower 95% CI	Upper 95% CI
Mean difference + 1.96 SD	30.332	11.897	48.768
Mean difference	1.170	-9.474	11.813
Mean difference - 1.96 SD	-27.993	-46.428	-9.558

propulsive impulse (N·s) [4jump] - propulsive impulse (N·s) [Impera]

Bias & Limits	Point Value	Lower 95% CI	Upper 95% CI
Mean difference + 1.96 SD	19.278	7.651	30.904
Mean difference	0.886	-5.826	7.599
Mean difference - 1.96 SD	-17.505	-29.132	-5.879

propulsive time (s) [4jump] - propulsive time (s) [Impera]

Bias & Limits	Point Value	Lower 95% CI	Upper 95% CI
Mean difference + 1.96 SD	0.012	0.005	0.019
Mean difference	6.000×10^{-4}	-0.003	0.005
Mean difference - 1.96 SD	-0.010	-0.018	-0.003

mean propulsive power relative to body mass (watt·kg^{-1}) [4jump] - mean propulsive power relative to body mass (watt·kg^{-1}) [Impera]

Bias & Limits	Point Value	Lower 95% CI	Upper 95% CI
Mean difference + 1.96 SD	1.425	0.580	2.271
Mean difference	0.088	-0.400	0.576
Mean difference - 1.96 SD	-1.249	-2.095	-0.404

Descriptive Statistics Squat JUMP (SJ)

Descriptive Statistics

	Mean (arithmetic)	95% Confidence Interval Mean		Std. Deviation
		Lower	Upper	
jump height (m) [4Jump]	0.282	0.251	0.313	0.044
jump height (m) [Impera]	0.289	0.259	0.319	0.042
RSI (AU) [4Jump]	0.918	0.752	1.083	0.232
RSI (AU) [Impera	0.947	0.787	1.106	0.223
propulsive average force (N) [4Jump]	962.423	841.029	1083.817	169.697
propulsive average force (N) [Impera]	954.590	829.408	1079.771	174.992
Impulso netto (N·s) [4Jump]	297.705	277.557	317.853	28.165
Impulso netto (N·s) [Impera]	293.621	271.853	315.389	30.429
propulsive time (s) [4Jump]	0.315	0.287	0.343	0.040
propulsive time (s) [Impera]	0.313	0.285	0.342	0.040
power relative to body mass (Watt·kg^{-1}) [4Jump]	12.693	10.609	14.777	2.913
power relative to body mass (Watt·kg^{-1}) [Impera]	12.786	10.621	14.951	3.026

Intraclass Correlation Squat jump (SJ)

***Intraclass Correlation* jump height (SJ).**

Type	Point Estimate	Lower 95% CI	Upper 95% CI
ICC2,1	0.885	0.625	0.970

Note. 10 subjects and 2 raters/measurements. ICC type as referenced by Shrout & Fleiss (1979).

***Intraclass Correlation* RSI$_{MOD}$ (SJ).**

Type	Point Estimate	Lower 95% CI	Upper 95% CI
ICC2,1	0.952	0.826	0.988

Note. 10 subjects and 2 raters/measurements. ICC type as referenced by Shrout & Fleiss (1979).

***Intraclass Correlation* propulsive average force (SJ).**

Type	Point Estimate	Lower 95% CI	Upper 95% CI
ICC2,1	0.976	0.911	0.994

Note. 10 subjects and 2 raters/measurements. ICC type as referenced by Shrout & Fleiss (1979).

***Intraclass Correlation* propulsive impulse (SJ).**

Type	Point Estimate	Lower 95% CI	Upper 95% CI
ICC2,1	0.871	0.585	0.966

Note. 10 subjects and 2 raters/measurements. ICC type as referenced by Shrout & Fleiss (1979).

***Intraclass Correlation* propulsive time (SJ).**

Type	Point Estimate	Lower 95% CI	Upper 95% CI
ICC2,1	0.991	0.967	0.998

Note. 10 subjects and 2 raters/measurements. ICC type as referenced by Shrout & Fleiss (1979).

***Intraclass Correlation* power relative to body mass (SJ).**

Type	Point Estimate	Lower 95% CI	Upper 95% CI
ICC2,1	0.985	0.943	0.996

Note. 10 subjects and 2 raters/measurements. ICC type as referenced by Shrout & Fleiss (1979).

Bland-Altman Plots Squat jump (SJ)

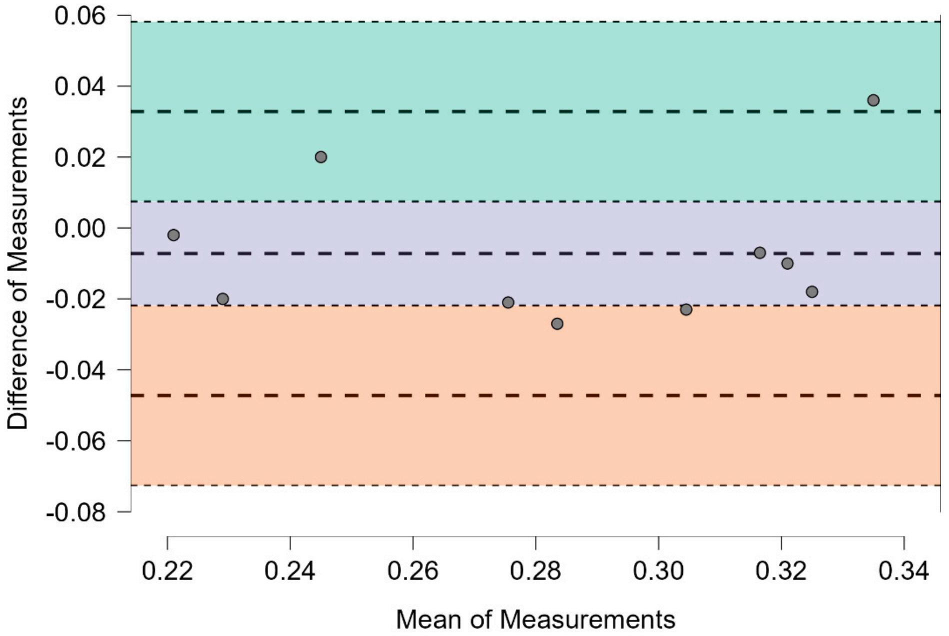

Figure S10. Bland–Altman plot for jump height (SJ).

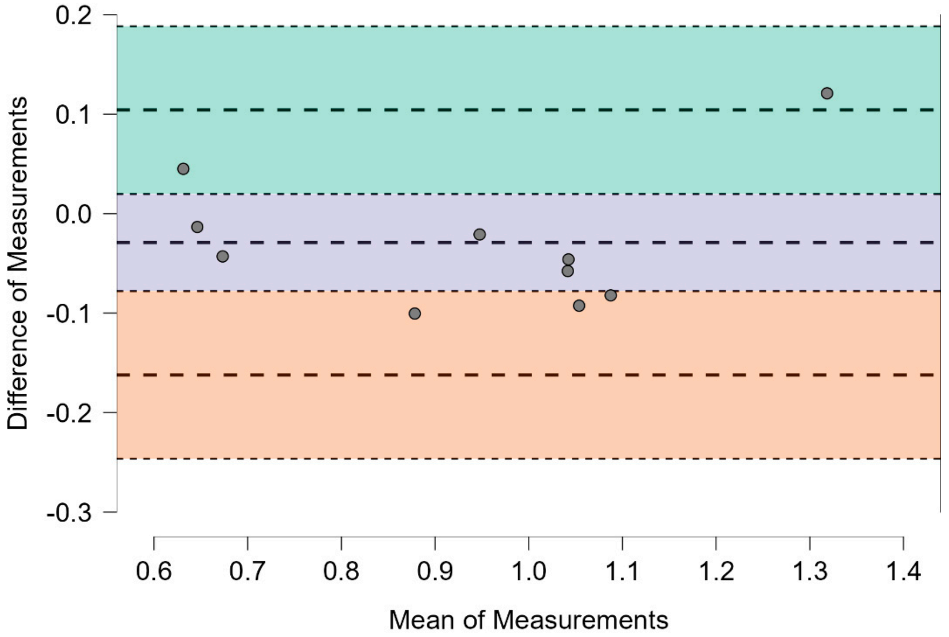

Figure S11. Bland–Altman plot for RSI_{MOD} (SJ).

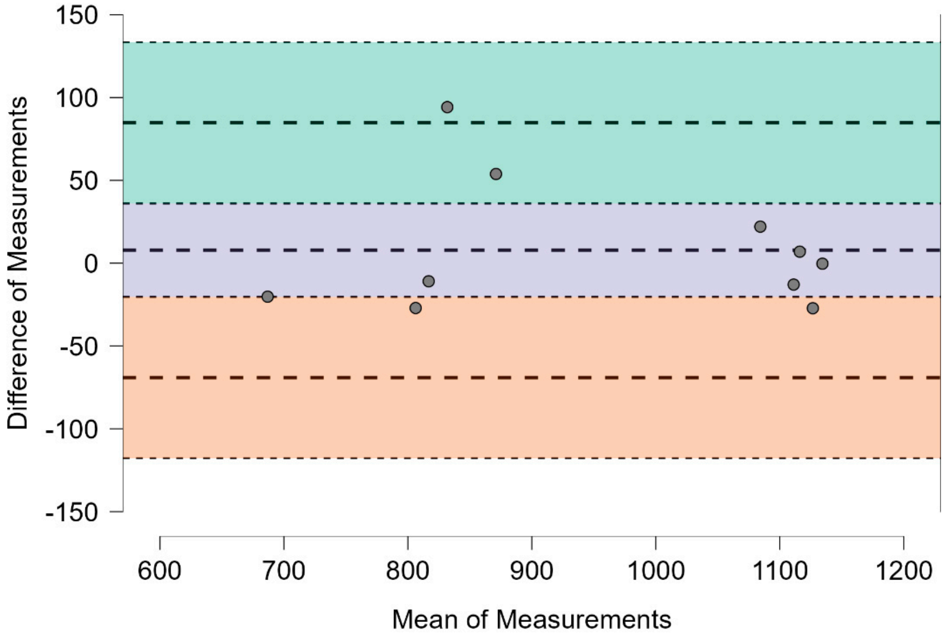

Figure S12. Bland–Altman plot for propulsive average force (SJ).

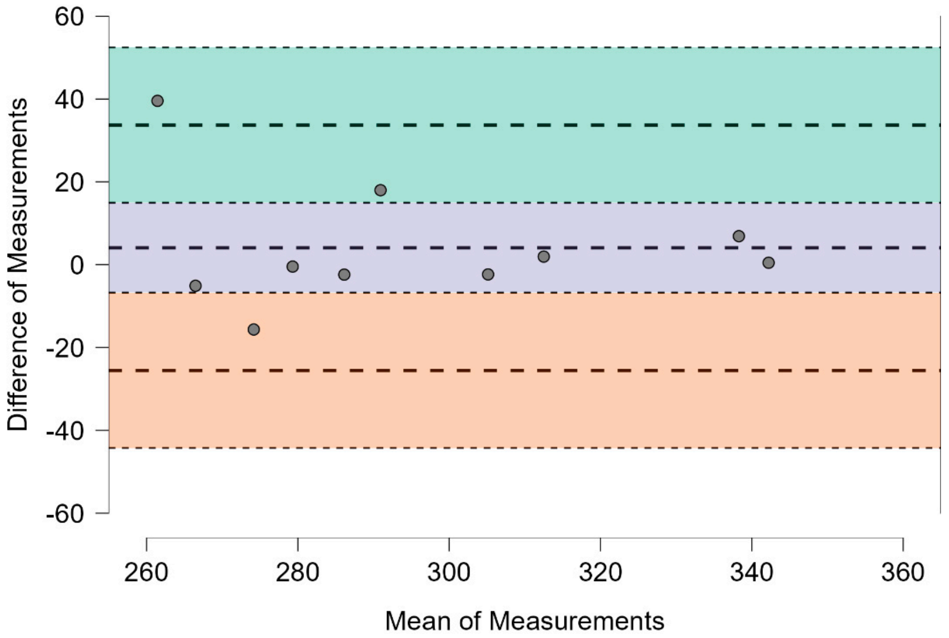

Figure S13. Bland–Altman plot for propulsive impulse (SJ).

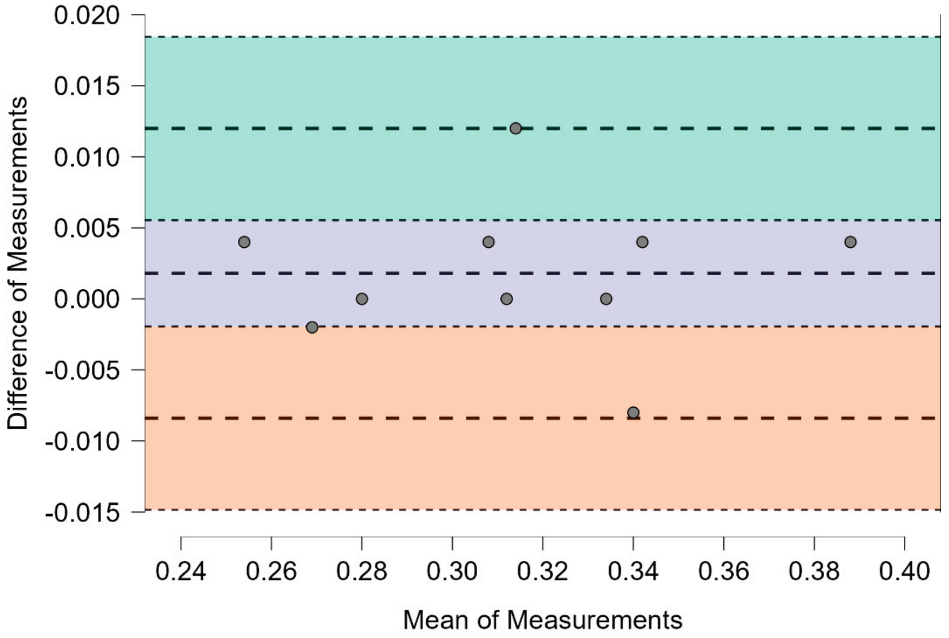

Figure S14. Bland–Altman plot for propulsive time (SJ).

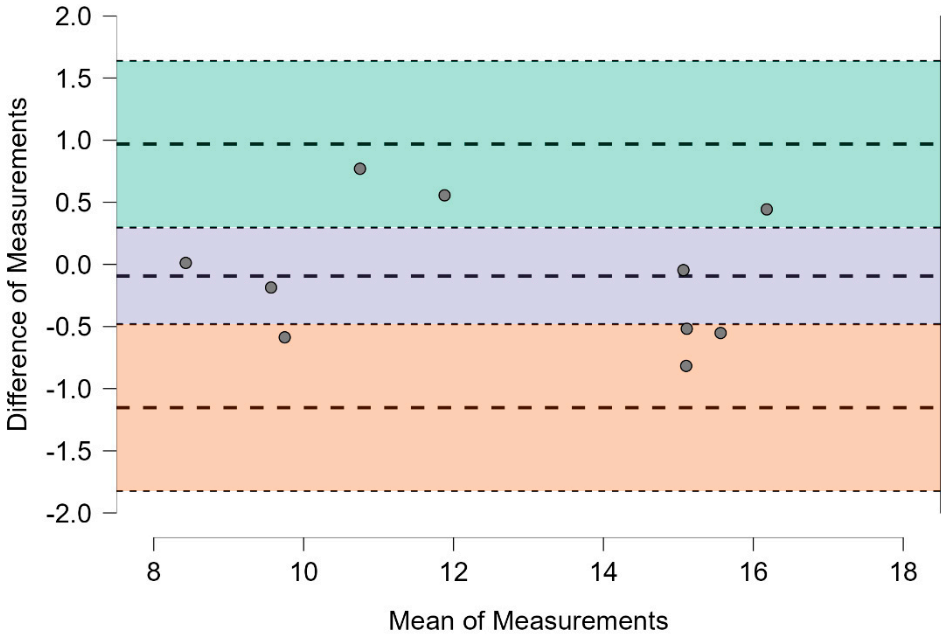

Figure S15. Bland–Altman plot for mean propulsive power relative to body mass (SJ).

Bland-Altman Tables Squat jump (SJ)

jump height (m) [4Jump] - jump height (m) [Impera]

Bias & Limits	Point Value	Lower 95% CI	Upper 95% CI
Mean difference + 1.96 SD	0.033	0.008	0.058
Mean difference	-0.007	-0.022	0.007
Mean difference - 1.96 SD	-0.047	-0.073	-0.022

RSI_{MOD} (AU) [4Jump] - RSI_{MOD} (AU) [Impera

Bias & Limits	Point Value	Lower 95% CI	Upper 95% CI
Mean difference + 1.96 SD	0.104	0.020	0.188
Mean difference	-0.029	-0.078	0.020
Mean difference - 1.96 SD	-0.162	-0.246	-0.078

propulsive average force (N) [4Jump] - propulsive average force (N) [Impera]

Bias & Limits	Point Value	Lower 95% CI	Upper 95% CI
Mean difference + 1.96 SD	84.746	36.125	133.367
Mean difference	7.833	-20.238	35.905
Mean difference - 1.96 SD	-69.079	-117.700	-20.458

propulsive impulse (N·s) [4Jump] - propulsive impulse (N·s) [Impera]

Bias & Limits	Point Value	Lower 95% CI	Upper 95% CI
Mean difference + 1.96 SD	33.702	14.979	52.425
Mean difference	4.084	-6.726	14.894
Mean difference - 1.96 SD	-25.534	-44.258	-6.811

propulsive time (s) [4Jump] - propulsive time (s) [Impera]

Bias & Limits	Point Value	Lower 95% CI	Upper 95% CI
Mean difference + 1.96 SD	0.012	0.006	0.018
Mean difference	0.002	-0.002	0.006
Mean difference - 1.96 SD	-0.008	-0.015	-0.002

Mean propulsive power relative to body mass (Watt·kg^{-1}) [4Jump] – mean propulsive power relative to body mass (Watt·kg^{-1}) [Impera]

Bias & Limits	Point Value	Lower 95% CI	Upper 95% CI
Mean difference + 1.96 SD	0.968	0.298	1.638
Mean difference	-0.093	-0.480	0.295
Mean difference - 1.96 SD	-1.153	-1.823	-0.483
